# Antidiabetic adiponectin receptor agonist AdipoRon suppresses tumour growth of pancreatic cancer by inducing RIPK1/ERK-dependent necroptosis

**DOI:** 10.1038/s41419-018-0851-z

**Published:** 2018-07-23

**Authors:** Miho Akimoto, Riruke Maruyama, Yasunari Kawabata, Yoshitsugu Tajima, Keizo Takenaga

**Affiliations:** 1 Department of Life Science, Shimane University Faculty of Medicine, 89-1 Ennya, Izumo, Shimane 693-8501 Japan; 2 Department of Pathology, Shimane University Faculty of Medicine, 89-1 Ennya, Izumo, Shimane 693-8501 Japan; 3 Department of Digestive and General Surgery, Shimane University Faculty of Medicine, 89-1 Ennya, Izumo, Shimane 693-8501 Japan; 4 Present Address: Department of Biochemistry, Teikyo University School of Medicine, 2-11-1 Kaga, Itabashi-ku, Tokyo, 173–8605 Japan; 5 Present Address: Laboratory of Cancer Genetics, Chiba Cancer Center Research Institute, 666-2 Nitona, Chiba, 260-8717 Japan

## Abstract

The association between lower circulating adiponectin (APN) levels and the development of pancreatic cancer has been reported. However, the effect of APN on the growth and survival of pancreatic cancer cells remains elusive. Here, we investigate the effects of the anti-diabetic APN receptor (AdipoR) agonist AdipoRon and APN on human pancreatic cancer cells. We found that AdipoRon, but not APN, induces MIAPaCa-2 cell death, mainly through necroptosis. Mechanistically, although both AdipoRon and APN activate AMPK and p38 MAPK in an AdipoR-dependent manner that elicits survival signals, only AdipoRon induces rapid mitochondrial dysfunction through mitochondrial Ca^2+^ overload, followed by superoxide production via RIPK1 and ERK1/2 activation. Oral administration of AdipoRon suppresses MIAPaCa-2 tumour growth without severe adverse effects and kills cancer cells isolated from patients with pancreatic cancer. Thus, AdipoRon could be a therapeutic agent against pancreatic cancer as well as diabetes.

## Introduction

Pancreatic cancer is quite notorious for its highly aggressive nature with chemotherapy-resistent and radiotherapy-resistant phenotypes and a poor prognosis. The incidence of pancreatic cancer is increasing annually worldwide, becoming the fourth most common cause of cancer-related death^1^. As the majority of pancreatic cancer patients are diagnosed at an inoperable stage^2,3^, typically chemotherapy and/or radiotherapy are the primary treatment modalities. However, even in patients receiving quality treatment, the overall 5-year relative survival rate is the lowest among cancer-related deaths. To survive such a dire situation, many efforts have been paid to improve local and systemic treatments clinically and to develop more effective and less toxic drugs.

Adiponectin (APN) is the most well-known adipokine exclusively secreted by adipose tissue^4–6^ and exhibits anti-diabetic, anti-atherogenic, anti-inflammatory and anti-angiogenic properties^7–9^. APN exerts its effects through the APN receptors AdipoR1 and AdipoR2^10,11^, activating intracellular cytoplasmic signalling molecules, including AMP-activated protein kinase (AMPK), p38 mitogen-activated protein kinase (p38 MAPK) and nuclear transcription factor peroxisome proliferators activated receptor α (PPARα)^9^. Animal studies have shown that APN enhances insulin sensitivity and ameliorates insulin resistance in animals^12,13^ and that circulating APN is inversely correlated with plasma insulin and is reduced in patients with obesity and type 2 diabetes mellitus^14^. Furthermore, plasma APN levels have been inversely associated with colorectal, endometrial and postmenopausal breast cancers^15–18^. With regard to pancreatic cancer development, the serum APN concentration is inversely correlated with rapid tumour growth in mice^19^. However, a genome-wide association study revealed that the nuclear receptor 5A2 (NR5A2) gene that activates the transcription of the APN gene is an important predisposing factor for pancreatic cancer^20^. Epidemiological data to date regarding circulating APN and pancreatic cancer risk have reported are inconsistent^21–26^. Furthermore, APN promotes pancreatic cancer progression by inhibiting apoptosis in murine Panc02-H7 and human Panc-1 cells^27^, whereas it contradictorily inhibits cell growth of Panc02 cells by inducing apoptosis^28^. Thus, the roles of APN in the development and growth of pancreatic cancer remain unclear.

AdipoRon is a synthetic small-molecule APN receptor agonist that binds to and stimulates both AdipoR1 and AdipoR2^29^. AdipoRon activates AMPK, p38 MAPK and PPARα pathways, improves insulin resistance and type 2 diabetes, and expands the shortened lifespan of db/db mice^29^. Notably, AdipoRon is the first orally active molecule and hence is expected to be applied clinically against a variety of conditions, including obesity, diabetes and cardiovascular disease. However, the effect of AdipoRon on the growth of pancreatic cancer cells has not been evaluated. In this study, we aimed to examine the effects of AdipoRon on the growth and survival of human pancreatic cancer cell lines and to compare the effects between AdipoRon and APN.

## Results

### AdipoRon induces cell death of pancreatic cancer cells

**Fig. 1:**
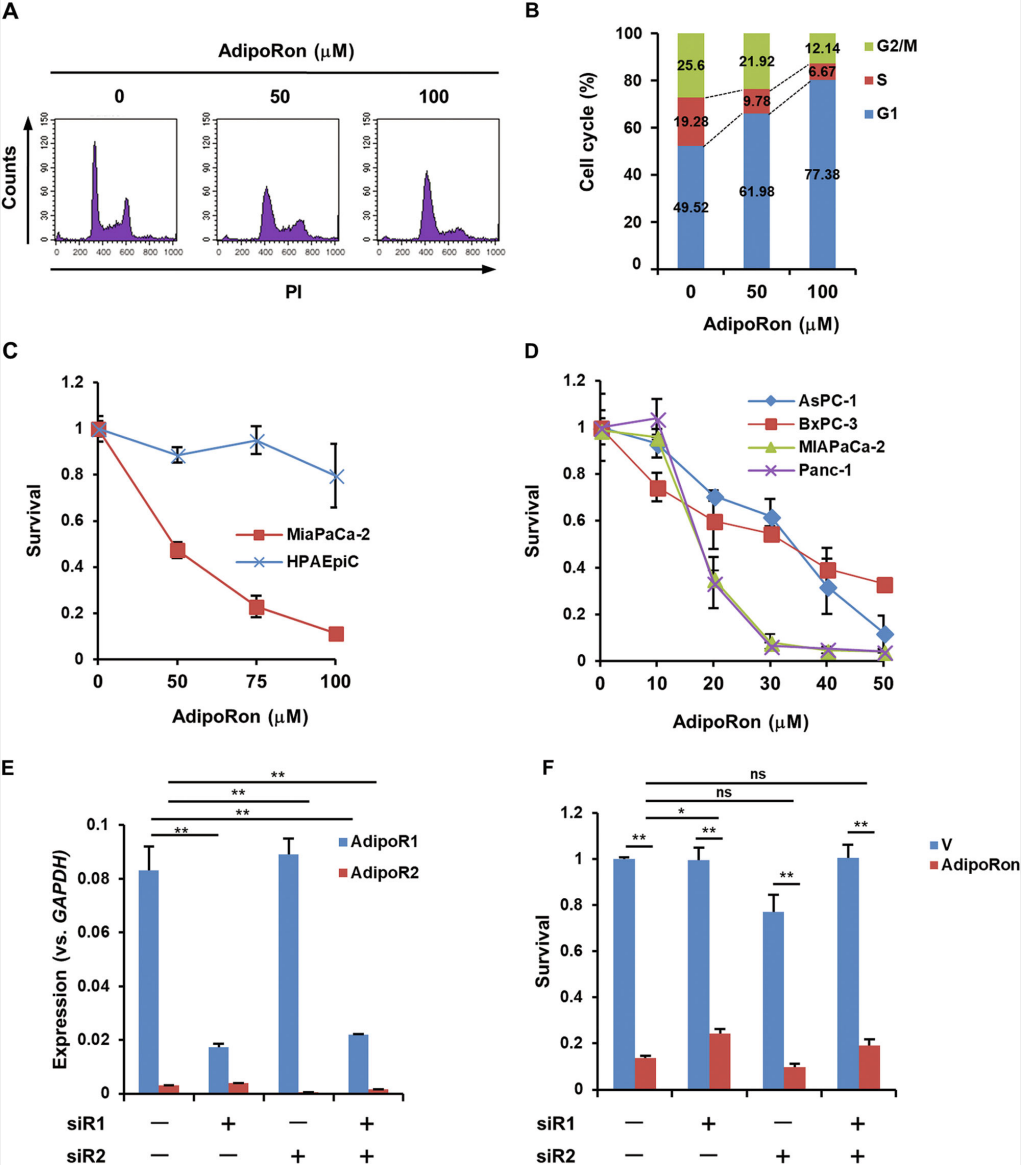
Effects of AdipoRon on the growth and viability of human pancreatic cancer cell lines. **a**, **b** Cell cycle progression of MIAPaCa-2 cells. The cells treated with solvent (DMSO) alone, 50 μM and 100 μM AdipoRon for 24 h were subjected to flow cytometric analysis. **c** Survival of MIAPaCa-2 cells and HPAEpiC cells. The cells were treated with various concentrations of AdipoRon for 40 h. **d** Survival of human pancreatic cancer cell lines. The cells were treated with various concentrations of AdipoRon for 6 days. **e** qRT-PCR analysis of the knockdown of AdipoR1 and AdipoR2 expression. **f** Effect of the knockdown of AdipoR1 and AdipoR2 expression on the survival of MIAPaCa-2 cells treated with solvent alone (V) or 100 μM AdipoRon for 40 h. For **c**–**f**, error bars represent standard deviation. *P < 0.05, **P < 0.001 by ANOVA test. ns not significant

We first evaluated the expression of AdipoRs in pancreatic cancer cell lines, normal epithelial HPAEpiC cells and human pancreatic cancer tissues. The results showed that all the examined cell lines preferentially expressed AdipoR1, and pancreatic cancer cell lines showed a higher level of AdipoR1 than normal epithelial HPAEpiC cells (Fig. S1A, B). Similar results were obtained in human pancreatic cancer tissues (Fig. S1C). Treatment of MIAPaCa-2 cells with AdipoRon arrested the cell cycle at G1/S phase (Fig. 1a, b) and subsequently induced death within 48 h. By contrast, AdipoRon only slightly reduced the viability of HPAEpiC cells (Fig. 1c). Treatment of AsPC-1, BxPC-3, MIAPaCa-2 and Panc-1 cells with lower doses of AdipoRon for 6 days also reduced cell growth and viability (Fig. 1d, Fig. S1A). To examine whether the cell death-inducing activity of AdipoRon is mediated by AdipoRs, we suppressed the expression of AdipoR1 and AdipoR2 by small-interfering RNAs (siRNAs) (Fig. 1e). Unexpectedly, knockdown of both AdipoR1 and AdipoR2 had only a marginal effect on AdipoRon-induced cell death (Fig. 1f).

### AdipoRon mainly induces RIPK1-dependent necroptosis in MIAPaCa-2 cells

**Fig. 2:**
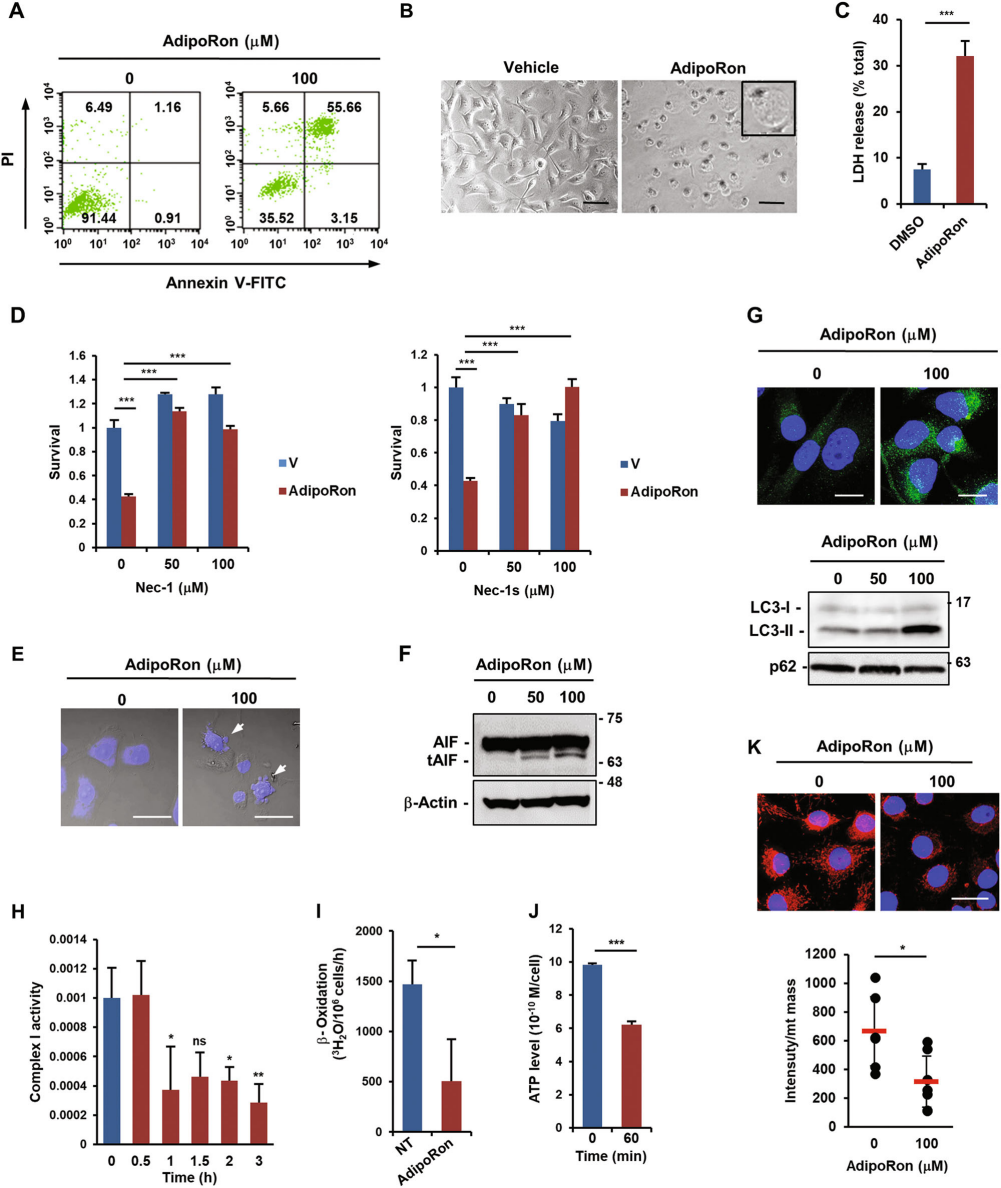
Effects of AdipoRon on death and the mitochondrial function of MIAPaCa-2 cells. **a** Annexin V/PI staining of AdipoRon-treated cells. The cells were treated with vehicle alone or 100 μM AdipoRon for 30 h. **b** Morphology of AdipoRon-treated cells. The cells were treated with vehicle alone or 100 μM AdipoRon for 40 h. Bars: 100 μm. **c** LDH release. The cells were treated with vehicle alone or 100 μM AdipoRon for 24 h. **d** Effects of Nec-1 and Nec-1s on AdipoRon-induced cell death. The cells were pre-treated with the drug for 1 h and then treated with vehicle alone (V) or 100 μM AdipoRon for 40 h in the presence of the drug. **e** DAPI staining of AdipoRon-treated cells. The cells were treated with 100 μM AdipoRon for 40 h. The arrows indicate fragmented nuclei. Bars: 100 µm. **f** Western blot analysis of AIF. The cells were treated with vehicle alone, 50 µM or 100 µM AdipoRon for 40 h. β-Actin was used as a loading control. **g** LC3 processing. The cells were treated with vehicle alone, 50 µM or 100 µM AdipoRon for 40 h. Upper: LC3 staining of the cells, lower: western blot of LC3 and p62. Full size images of the western blots presented are shown in Figure S13. **h** Complex I activity. The cells were treated with various concentrations of AdipoRon for the indicated times. **i** β-oxidation. The cells were treated with vehicle alone or 100 μM AdipoRon for 1 h. **j** ATP production. The cells were treated with vehicle alone or 100 μM AdipoRon for 1 h. **k** Mitochondrial membrane potential. Upper: the cells were treated with vehicle alone or 100 μM AdipoRon for 4 h and stained with MitoTracker Red CMXRos. The cells were fixed and stained with DAPI. Lower: semi-quantification of relative fluorescence intensity. The pixel values of MitoSOX Red and mitochondrial mass (area) were calculated for each cell to determine the relative fluorescence intensity using the ImageJ software. Bar: 20 μm. For **c**, **d**, **h**–**k** error bars represent standard deviation. *P < 0.05, **P < 0.01, ***P < 0.001 by Student’s *t*-test or ANOVA test. ns not significant

Treatment of MIAPaCa-2 cells with AdipoRon increased the number of annexin V^+^/PI^+^ cells (Fig. 2a). Because annexin V^+^/PI^+^ cells could represent either late apoptotic cells or necrotic cells^30^, we tested the effect of pan-caspase inhibitor Z-VAD-FMK to determine the type of cell death and found that it did not ameliorate AdipoRon-induced cell death (Fig. S2A). Moreover, AdipoRon neither increased caspase-3/9 activities nor altered the expression of apoptosis-related genes (Fig. S2B, C), thus excluding caspase-dependent apoptosis. Morphologically, AdipoRon induced cytoplasmic swelling with large bubbles blowing from the plasma membrane in almost all cells (Fig. 2b), demonstrating a disruption of the osmotic potential, which was confirmed by LDH release (Fig. 2c). Curiously, although MIAPaCa-2 cells lack the RIPK3 that is required for classical necroptosis^31^, the necroptosis inhibitors with RIPK1 inhibitory activity, necrostatin-1 (Nec-1) and necrostatin-1s (Nec-1s)^32^, blocked AdipoRon-induced cell death (Fig. 2d). On the basis of these results, cell death was likely to be RIPK1-dependent, RIPK3-independent necroptosis. However, a close examination of the nucleus revealed a mild fragmentation, a feature of apoptosis, in some cells (Fig. 2e). In fact, we detected a small amount of the truncated form of apoptosis-inducing factor (tAIF), which is involved in caspase-independent apoptosis^33^, in AdipoRon-treated cells (Fig. 2f). In addition, AdipoRon slightly induced autophagy, as evaluated by the appearance of LC3 puncta, an increase in the LC3-II/LC3-I ratio and a slight decrease in p62 protein (Fig. 2g). The autophagy inhibitor chloroquine partially prevented cell death (Fig. S2D). Therefore, autophagy might also be involved in cell death. Ferrostatin-1 (Ferr-1), a ferroptosis inhibitor^34^, showed no effect on AdipoRon-induced cell death (Fig. S2E). Remarkably, we noticed that AdipoRon caused mitochondrial dysfunction within a few hours, as revealed by the reductions of mitochondrial complex I activity, β-oxidation and ATP production (Fig. 2h–j), followed by decreased mitochondrial membrane potential (Fig. 2k). Taken together, these data demonstrated that AdipoRon-treated MIAPaCa-2 cells died largely via RIPK1-dependent necroptosis along with small fractions of caspase-independent apoptosis and autophagic cell death caused by rapid mitochondrial dysfunction.

### AdipoRon induces a rapid mitochondrial Ca^2+^ overload followed by superoxide production in MIAPaCa-2 cells

**Fig. 3:**
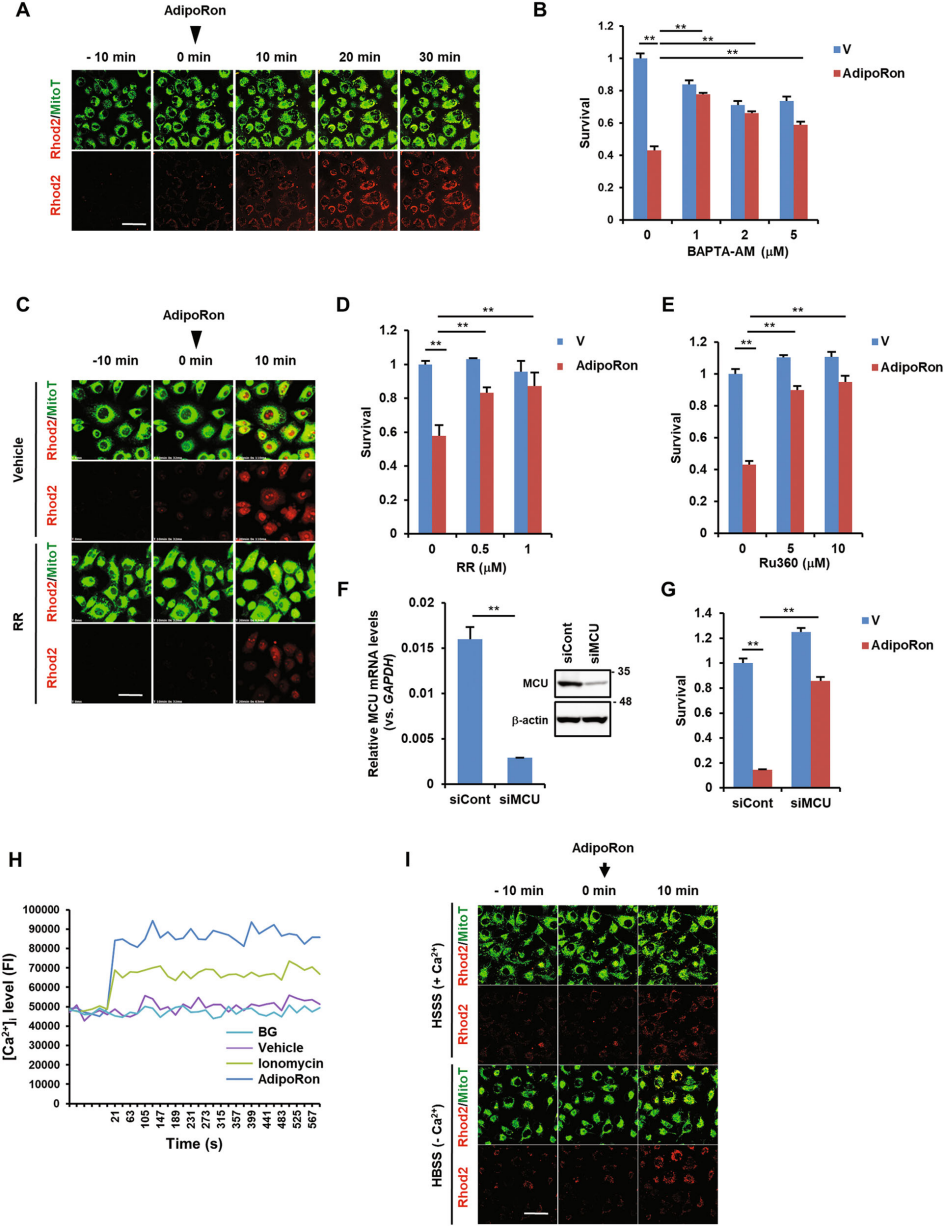
Involvement of [Ca^2+^]_i_ and [Ca^2+^]mt in AdipoRon-induced cell death in MIAPaCa-2 cells. **a** [Ca^2+^]mt level. Time-lapse imaging was used to evaluate [Ca^2+^]mt levels. The cells were loaded with Rhod2-AM and MitoTracker Green (MitoT). AdipoRon (100 μM) was added at 0 min. Bar: 50 μm. **b** Effect of BAPTA-AM on AdipoRon-induced cell death. The cells were pre-treated with BAPTA-AM for 1 h and then treated with vehicle alone (V) or 100 μM AdipoRon for 40 h in the presence of BAPTA-AM. **c** Effect of RR on AdipoRon-induced [Ca^2+^]mt accumulation. Cells loaded with Rhod2-AM and MitoTracker Green (MitoT) were treated with 100 μM AdipoRon in the presence or absence of 1 μM RR. AdipoRon (100 μM) was added at 0 min. Bar: 50 μm. **d** Effect of ruthenium red (RR) on AdipoRon-induced cell death. The cells were pre-treated with RR for 1 h and then treated with vehicle alone (V) or 100 μM AdipoRon for 40 h in the presence of RR. **e** Effect of Ru360 on AdipoRon-induced cell death. The cells were pre-treated with Ru360 for 1 h and then treated with vehicle alone (V) or 100 μM AdipoRon for 20 h in the presence of Ru360. **f** Downregulation of MCU by siRNA. Left: qRT-PCR. Right: western blot. Full size images of the western blots presented are shown in Figure S13. **g** Effect of knockdown of MCU on AdipoRon-induced cell death. The cells transfected with control siRNA (siCont) or siMCU were incubated with vehicle alone or 100 μM AdipoRon for 20 h. **h** [Ca^2+^]_i_ level. The cells loaded with Fluo4-AM were treated with vehicle alone or 100 μM AdipoRon. Ionomycin (1 μM) was used as a positive control. BG: background. **i** Effect of extracellular Ca^2+^ on [Ca^2+^]mt levels. The cells loaded with Rhod2-AM and MitoTracker Green (MitoT) were treated with 100 μM AdipoRon in Ca^2+^-free HBSS with or without 1 mM CaCl_2_. AdipoRon (100 μM) was added at 0 min. Bar: 50 μm. For **b**, **d**–**g**, error bars represent standard deviation. *P < 0.03, **P < 0.001 by Student’s *t*-test or ANOVA test

To investigate the mechanism underlying AdipoRon-induced mitochondrial dysfunction, we focused on mitochondrial Ca^2+^ ([Ca^2+^]mt) because it is closely related to cell death^35,36^. We examined [Ca^2+^]mt using the cell-permeable fluorescent Ca^2+^ indicator Rhod2-AM^37^. Notably, [Ca^2+^]mt level increased shortly after the addition of AdipoRon (Fig. 3a, Fig. S3). As the intracellular Ca^2+^ chelator BAPTA-AM attenuated AdipoRon-induced cell death (Fig. 3b) and the non-specific calcium channel blocker ruthenium red abolished AdipoRon-induced [Ca^2+^]mt influx and cell death (Fig. 3c, d, Fig. S3), the increase in [Ca^2+^]mt through calcium channels was responsible for the cell death. Because it is known that [Ca^2+^]mt uptake is mediated by mitochondrial Ca^2+^ uniporter (MCU)^38,39^, we examined the effect of the MCU inhibitor Ru360 and found that it blocked AdipoRon-induced cell death (Fig. 3e). Furthermore, siRNA-mediated downregulation of MCU protected the cells from death (Fig. 3f, g). The increase in [Ca^2+^]mt seemed to follow an increase in intracellular Ca^2+^ ([Ca^2+^]_i_) because the use of Fluo4-AM as a probe revealed a rise in the [Ca^2+^]_i_ level within a few minutes after the addition of AdipoRon (Fig. 3h). Knockdown of AdipoRs marginally influenced the [Ca^2+^]_i_ level (Fig. S4), suggesting that the effect of AdipoRon on [Ca^2+^]_i_ flux occurred independently of AdipoRs. To identify the source of Ca^2+^, extracellular or endoplasmic reticulum (ER), we treated the cells with AdipoRon with or without extracellular Ca^2+^ and found that the level of [Ca^2+^]mt was increased under both conditions (Fig. 3i, Fig. S3), indicating the possible involvement of ER Ca^2+^ stores. As expected, the plasma membrane L-type channel blocker nifedipine and the T-type channel blocker mibefradil showed no effect on AdipoRon-induced cell death (Fig. S5A, B). By contrast, while the ER IP3R/Ca^2+^ channel antagonist 2-APB did not affect AdipoRon-induced cell death, the ryanodine receptor (RyR) channel inhibitor dantrolene slightly inhibited cell death (Fig. S5C, D). Collectively, these results suggested that a rise in [Ca^2+^]_i_ release from ER, possibly through RyR followed by Ca^2+^ overload in mitochondria through MCU, was crucial for AdipoRon-induced cell death.

It has recently been shown that MCU activity is regulated by a Ca^2+^-dependent and ROS-dependent proline-rich tyrosine kinase 2 (Pyk2) that directly phosphorylates MCU and enhances [Ca^2+^]mt uptake by promoting MCU channel oligomerization and the formation of tetrameric channels or by Ca^2+^/calmodulin-dependent protein kinase II (CaMKII), although its role is still controversial^39^. However, in MIAPaCa-2 cells, neither the Pyk2/FAK inhibitor PF-431396 nor the CaMKII inhibitor KN-93 prevented AdipoRon-induced cell death (Fig. S6A, B), excluding the involvement of Pyk2 and CaMKII.

**Fig. 4:**
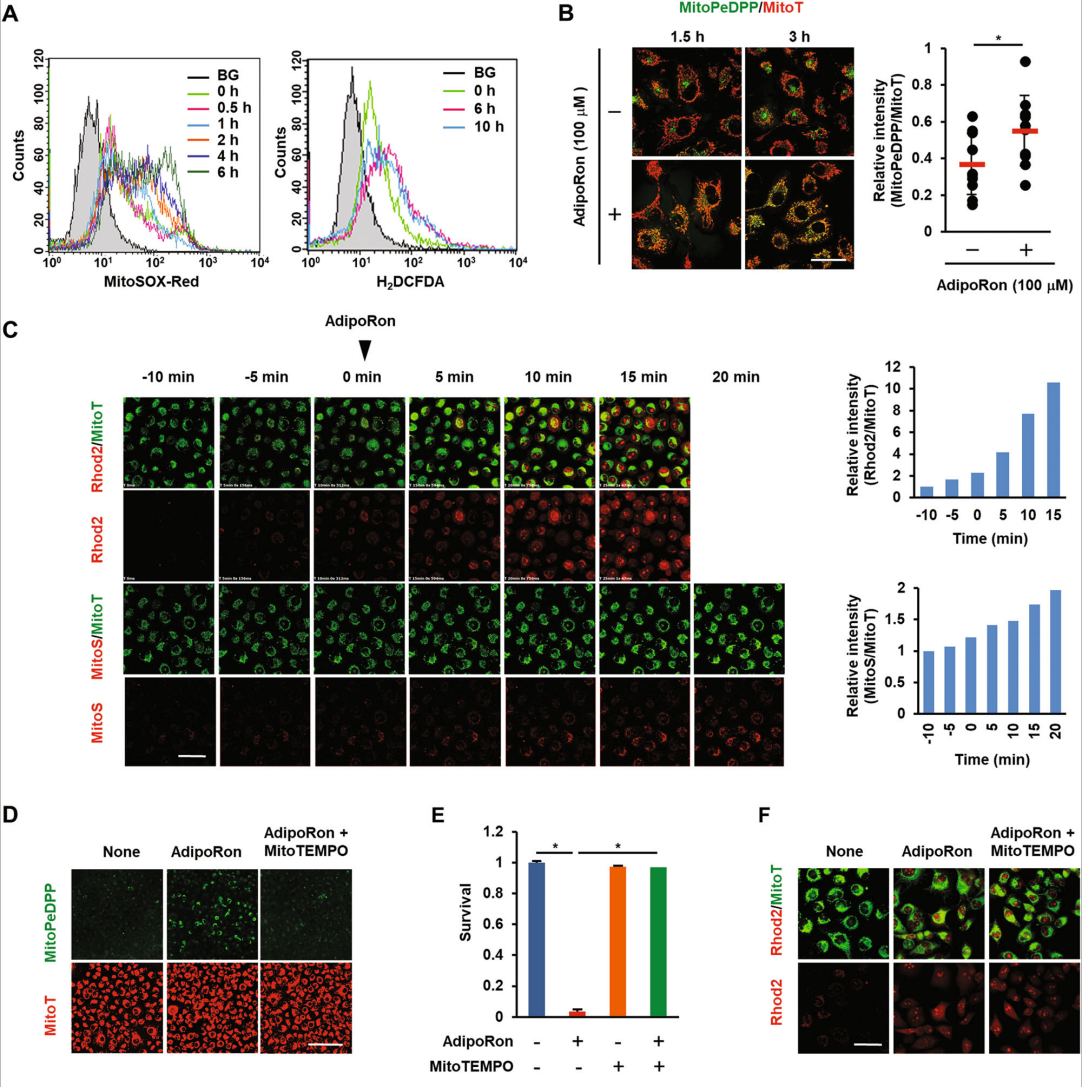
Effects of AdipoRon on mtROS production, lipid peroxidation and [Ca^2+^]mt level in MIAPaCa-2 cells. **a** Mitochondrial and intracellular ROS production. The cells were treated with vehicle alone or 100 μM AdipoRon for the indicated times. Mitochondrial ROS and intracellular ROS were examined by FACS after staining the cells with MitoSOX Red and H_2_DCF-DA, respectively. BG: background. **b** Mitochondrial lipid peroxidation. The cells were treated with vehicle alone or 100 μM AdipoRon for 1.5 h or 3 h. Lipid peroxidation was evaluated by staining the cells with MitoPeDPP. Bar: 50 μm. The pixel values of MitoPeDPP and MitoT on 3 h-images were calculated for each cell to determine the relative fluorescence intensity using the ImageJ software. **c** [Ca^2+^]mt level. Time-lapse imaging was used to evaluate [Ca^2+^]mt and mtROS levels. For [Ca^2+^]mt, the cells were loaded with Rhod2-AM and MitoTracker Green (MitoT). For mtROS, the cells were loaded with MitoSOX Red (MitoS) and MitoTracker Green (MitoT). AdipoRon (100 μM) was added at 0 min. Bar: 50 μm. The pixel values of Rhod2 or MitoS and MitoT were calculated for each cell to determine the relative fluorescence intensity using the ImageJ software. **d** Effect of MitoTEMPO on AdipoRon-induced lipid peroxidation. The cells treated with 100 μM AdipoRon in the absence or presence of 10 μM MitoTEMPO for 3 h were stained with MitoPeDPP and MitoTracker Red (MitoT). Bar: 500 μm. **e** Effect of MitoTEMPO on AdipoRon-induced cell death. The cells were treated with 100 μM AdipoRon in the absence or presence of 10 μM MitoTEMPO for 40 h. **f** Effect of MitoTEMPO on AdipoRon-induced [Ca^2+^]mt accumulation. The cells pre-treated with 10 μM MitoTEMPO for 1 h were loaded with Rhod-2AM and MitoTracker Green (MitoT). The images were taken 10 min after the addition of AdipoRon (100 μM). Bar: 50 μm. For **e**, error bars represent standard deviation. *P < 0.001 by ANOVA test

Mitochondrial Ca^2+^ overload has been shown to enhance mitochondrial superoxide production (hereafter referred to as mtROS)^40^. We then monitored mtROS production with MitoSOX Red. Intracellular ROS were also measured with H_2_DCF-DA. FACS analysis revealed that mtROS production was detectable as early as 1 h after the addition of AdipoRon. Intracellular ROS was slightly increased as early as 1 h (Fig. S7) and significantly increased by 6 h (Fig. 4a). Along with mtROS production, an increase in lipid peroxidation of mitochondrial membrane was evident within 1.5 h (Fig. 4b). When observed under a confocal microscope, mtROS production was found to increase as early as 15 min after the addition of AdipoRon and was preceded by the increase in the [Ca^2+^]mt level (Fig. 4c). Importantly, preincubation of the cells with the mitochondria-targeted antioxidant MitoTEMPO ameliorated lipid peroxidation and cell death induced by AdipoRon (Fig. 4d, e, Fig. S3). MitoTEMPO had virtually no effect on the [Ca^2+^]mt level (Fig. 4f, Fig. S3), consistent with the observation that [Ca^2+^]mt is upstream of mtROS production^40^. Thus, these results indicated that [Ca^2+^]mt overload followed by mtROS production could be attributed to AdipoRon-induced mitochondrial dysfunction and cell death.

### Signalling pathways in AdipoRon-induced cell death

**Fig. 5:**
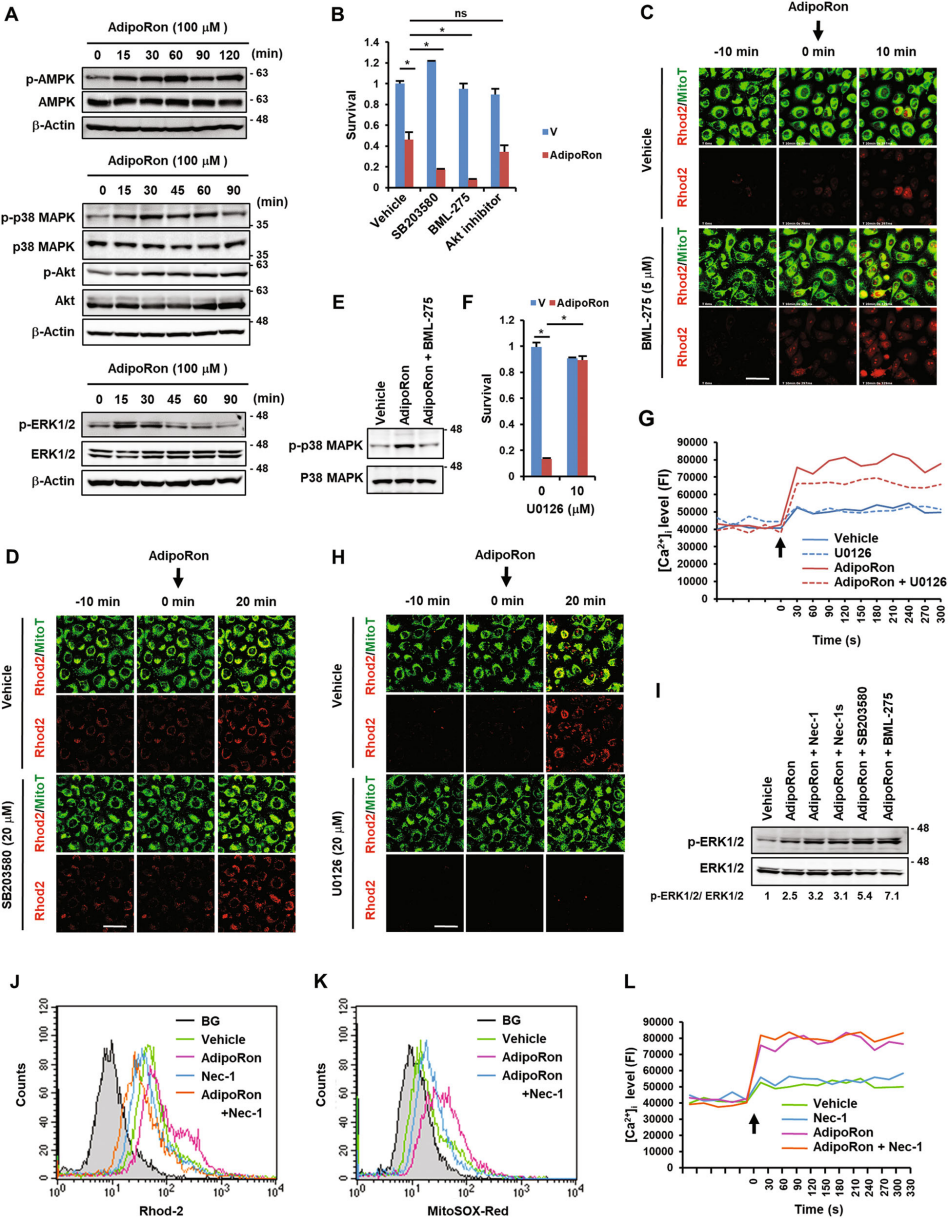
Analyses of signalling pathways of AdipoRon-induced cell death in MIAPaCa-2 cells. **a** Western blot analyses of the activation of AMPK, p38 MAPK, Akt and ERK1/2 by AdipoRon. The cells were treated with 100 μM AdipoRon for the indicated time periods. **b** Effects of various inhibitors on AdipoRon-induced cell death. The cells were pre-treated with vehicle alone (V) or the drug for 1 h and then with 100 μM AdipoRon in the presence or absence of the drug (20 μM SB203580, 5 μM BML-275 or 1 μM Akt inhibitor) for 40 h. **c** Effect of BML-275 on AdipoRon-induced [Ca^2+^]mt accumulation. The cells pre-treated with 5 μM BML-275 for 1 h were loaded with Rhod2-AM and MitoTracker Green (MitoT). Vehicle alone or 100 μM AdipoRon was added at time 0. Bar: 50 μm. **d** Effect of SB203580 on AdipoRon-induced [Ca^2+^]mt accumulation. The cells pre-treated with 20 μM SB203580 for 1 h were loaded with Rhod2-AM and MitoTracker Green (MitoT). Vehicle alone or 100 μM AdipoRon was added at time 0. Bar: 50 μm. **e** Effect of BML-275 on AdipoRon-induced phosphorylation of p38 MAPK. **f** Effect of U0126 on AdipoRon-induced cell death. The cells were pre-treated with vehicle alone (V) or 10 μM U0126 for 1 h and then treated with 100 μM AdipoRon in the presence or absence of 10 μM U0126 for 40 h. **g** Effect of U0126 on AdipoRon-induced [Ca^2+^]_i_ accumulation. The cells were loaded with Fluo4-AM. Vehicle alone or 100 μM AdipoRon or 20 μM U0126 was added at time 0. **h** Effect of U0126 on AdipoRon-induced [Ca^2+^]mt accumulation. Cells loaded with Rhod2-AM and MitoTracker Green (MitoT) were treated with 100 μM AdipoRon in the presence or absence of 20 μM U0126. Bar: 50 μm. **i** Effect of various inhibitors on AdipoRon-induced ERK1/2 phosphorylation. The cells were pre-treated with 50 μM Nec-1, 50 μM Nec-1s, 20 μM SB203580 and 5 μM BML-275 for 1 h, and then with 100 μM AdipoRon in the presence or absence of the inhibitors for 30 min. The ratio of p-ERK1/2 to ERK1/2 is shown below. **j** Effect of Nec-1 on AdipoRon-induced [Ca^2+^]mt accumulation. The cells loaded with Rhod2-AM were treated with 100 μM AdipoRon in the presence or absence of 100 μM Nec-1 for 6 h. BG: background. **k** Effect of Nec-1 on AdipoRon-induced mtROS generation. The cells loaded with MitoSOX Red were treated with 100 μM AdipoRon in the presence or absence of 50 μM Nec-1 for 8 h. BG: background. (**l**) Effect of Nec-1 on AdipoRon-induced [Ca^2+^]_i_ accumulation. The cells loaded with Fluo4-AM were treated with 100 μM AdipoRon in the presence or absence of 50 μM Nec-1. For **b** and **f**, error bars represent standard deviation. *P < 0.001 by Student’s *t*-test or ANOVA test. ns not significant. Full size images of the western blots presented are shown in Figure S13

We found that AdipoRon induced sustained phosphorylation of AMPK, p38 MAPK and Akt, and transient phosphorylation of ERK1/2 (p44/42 MAPK), all of which were detectable within 15 min after treatment (Fig. 5a). Incubation of the cells with the AMPK inhibitor BML-275 (Compound C) and the p38 MAPK inhibitor SB203580 further potentiated AdipoRon-induced cell death, with the former being more potent than the latter (Fig. 5b). The Akt inhibitor showed no effect (Fig. 5b). Correspondingly, BML-275 further enhanced an early increase in [Ca^2+^]mt influx induced by AdipoRon (Fig. 5c, Fig. S3). Knockdown of AMPK by siRNA showed similar effects (Fig. S8). SB203580 also enhanced, although to a lesser degree than BML-275, [Ca^2+^]mt levels induced by AdipoRon (Fig. 5d, Fig. S3). These results suggested that both AMPK and p38 MAPK generated survival signals and had roles upstream of the [Ca^2+^]mt influx. It should be noted that BML-275 inhibited the phosphorylation of p38 MAPK induced by AdipoRon (Fig. 5e), indicating that p38 MAPK was localized downstream of AMPK.

We also found that the MEK inhibitor U0126 alleviated AdipoRon-induced cell death (Fig. 5f). Correspondingly, U0126 suppressed AdipoRon-enhanced [Ca^2+^]_i_ and [Ca^2+^]mt levels (Fig. 5g, h, Fig. S3), indicating that ERK1/2 activation led to increases in [Ca^2+^]_i_ and [Ca^2+^]mt levels and subsequently cell death. Interestingly, both BML-275 and SB203580 enhanced AdipoRon-induced ERK1/2 activation (Fig. 5i), indicating that both AMPK and p38 MAPK activation suppressed ERK1/2 activation. These results indicated that ERK1/2 generated a death signal by increasing [Ca^2+^]_i_ and [Ca^2+^]mt influx, which was suppressed by the activation of AMPK and p38 MAPK.

We also examined the possible involvement of the AdipoR2-PPARα pathway^41,42^. The PPARα agonist ciprofibrate protected the cells from AdipoRon-induced cell death, and conversely, the PPARα antagonist GW6471 further enhanced cell death (Fig. S9A, B). The PPARγ agonist rosiglitazone, but not the PPARβ agonist GW50156, blocked, whereas the PPARγ antagonist GW9662 enhanced, cell death (Fig. S9A, C). Therefore, direct and potent activation of PPARα/γ by the agonists could rescue the cells from AdipoRon-induced cell death. Activation of PPARα/γ has been reported to stimulate the transcription of antioxidant-detoxifying enzyme genes, including catalase (CAT), glutathione peroxidase 3 (GPx3), superoxide dismutase 1 (SOD1), SOD2 and uncoupling protein 2 (UCP2)^43,44^. In fact, we found that AdipoRon slightly and gradually enhanced the expression of the genes encoding CAT, GPx3, SOD1 and UCP2, but not SOD2 (Fig. S9D). These results indicate that the PPARα/γ-antioxidant genes axis is potentially active to eliminate ROS in the cells. However, because AdipoR2 knockdown showed no effect on AdipoRon-induced cell death, the activity of AdipoR2-PPARα/γ pathway may be too weak to ameliorate AdipoRon-induced cell death.

Notably, Nec-1 reduced the AdipoRon-induced increase in [Ca^2+^]mt and mtROS generation (Fig. 5j, k), but not in [Ca^2+^]_i_ (Fig. 5l). Therefore, it is likely that RIPK1 stimulated Ca^2+^ influx into mitochondria. However, we found that Nec-1 slightly augmented AdipoRon-induced ERK1/2 phosphorylation (Fig. 5i), indicating that RIPK1 also showed a weak ability to suppress ERK1/2 activation. These results suggested that the ability of RIPK1 to stimulate the AdipoRon-induced [Ca^2+^]mt influx might be stronger than that needed to suppress ERK1/2 phosphorylation. By contrast, Nec-1 did not show any effect on the phosphorylation of AMPK and p38 MAPK enhanced by AdipoRon (Fig. S10).

### Adiponectin slightly inhibits proliferation but does not induce death of MIAPaCa-2 cells

**Fig. 6:**
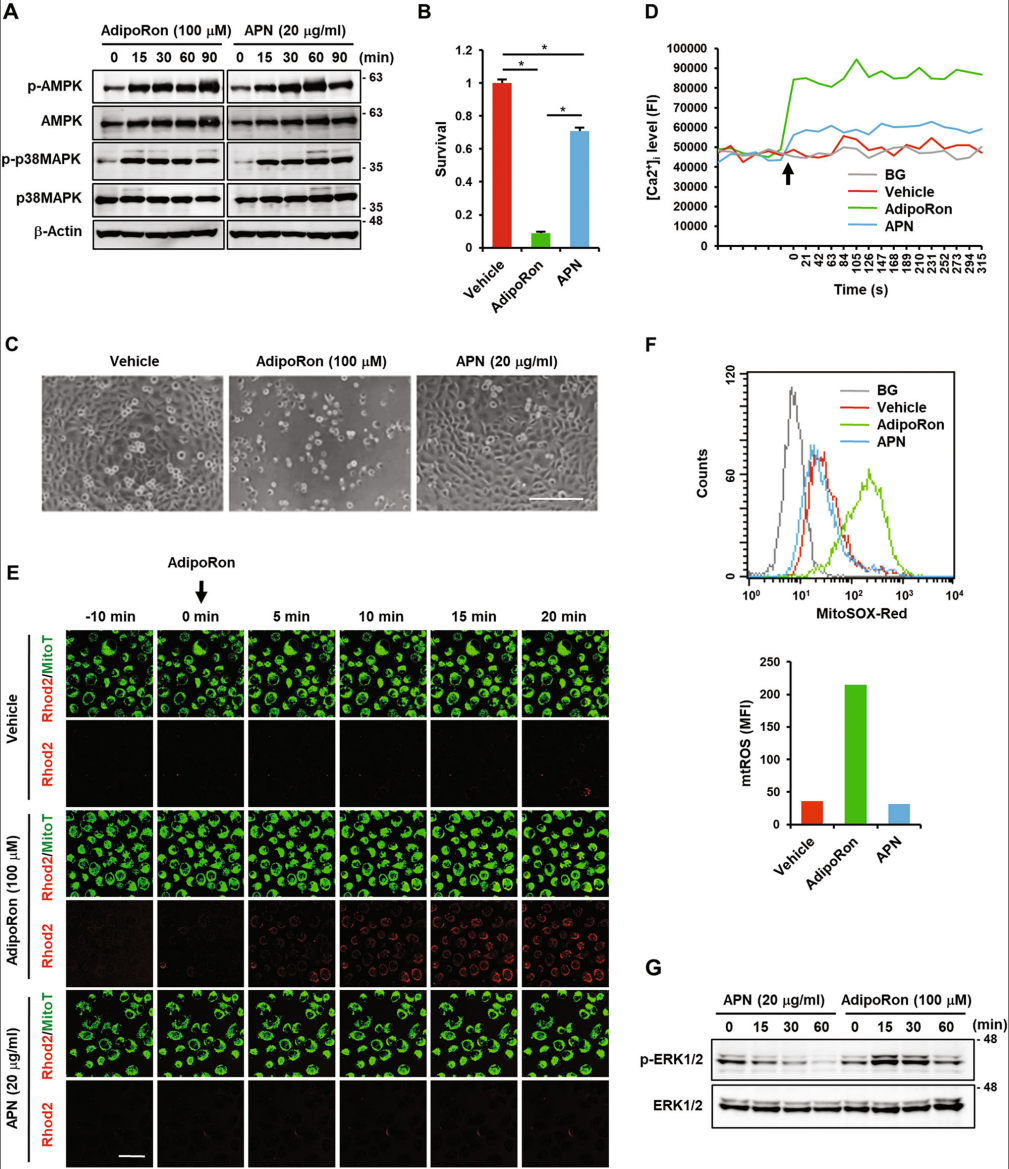
Effects of APN on MIAPaCa-2 cells. **a** Effects of AdipoRon and APN on the phosphorylation of AMPK and p38 MAPK. The cells were treated with 100 μM AdipoRon or 20 μg/ml APN for the indicated time periods. β-Actin was used as a loading control. **b** Effects of AdipoRon and APN on survival. The cells were treated with 100 μM AdipoRon or 20 μg/ml APN for 40 h. **c** Morphology of the cells treated with vehicle alone, 100 μM AdipoRon or 20 μg/ml APN for 40 h. Bar: 200 μm. **d** Effects of AdipoRon and APN on [Ca^2+^]_i_ accumulation. The cells loaded with Fluo4-AM were treated with 100 μM AdipoRon or 20 μg/ml APN. Ionomycin (1 μM) was used as a positive control. BG: background. **e** Effects of AdipoRon and APN on [Ca^2+^]mt accumulation. The cells loaded with Rhod2-AM and MitoTracker Green were treated with 100 μM AdipoRon or 20 μg/ml APN. Bar: 50 μm. **f** Effects of AdipoRon and APN on mtROS generation. The cells loaded with MitoSOX Red were treated with 100 μM AdipoRon or 20 μg/ml APN. BG: background. **g** Effects of AdipoRon and APN on ERK1/2 phosphorylation. The cells were treated with 100 μM AdipoRon or 20 μg/ml APN for the indicated time periods. β-Actin was used as a loading control. For **b**, error bars represent standard deviation. *P < 0.001 by ANOVA test. Full size images of the western blots presented are shown in Figure S13

We next examined the effect of APN on the growth and death of MIAPaCa-2 cells. Because AdipoR activation led to the phosphorylation of AMPK and p38 MAPK (Fig. 5a), we used APN at a concentration of 20 μg/ml, at which AMPK and p38 MAPK were activated to the same extent as AdipoRon (Fig. 6a). The results showed that although APN slightly inhibited growth, it did not induce cell death (Fig. 6b, c). APN weakly increased [Ca^2+^]_i_ and did not increase [Ca^2+^]mt and mtROS to detectable levels (Fig. 6d–f, Fig. S3). Furthermore, APN suppressed the basal level of ERK1/2 phosphorylation (Fig. 6g), probably through activation of AMPK and p38 MAPK. These data indicated that APN activated survival signalling pathways and simultaneously inhibited the death signalling pathway.

### AdipoRon inhibits tumour growth of MIAPaCa-2 cells

**Fig. 7:**
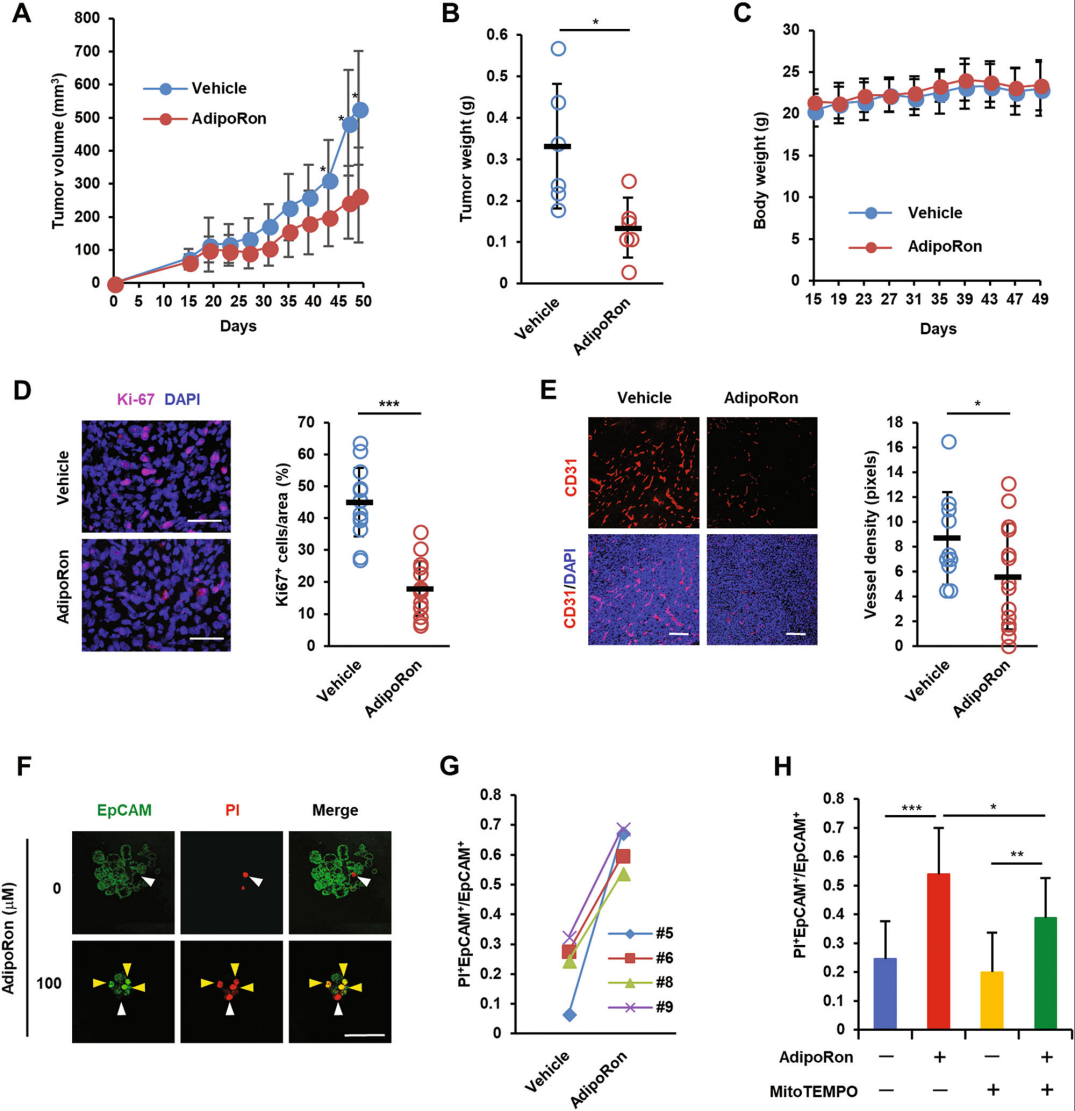
Antitumor effect of AdipoRon. **a** Effect of oral administration of AdipoRon on the growth of subcutaneous MIAPaCa-2 tumours. Twelve days after subcutaneous implantation of MIAPaCa-2 cells (1 × 10^6^ cells), vehicle alone or AdipoRon (60 mg/kg) were orally administered every other day (*n* = 6 per group). **b** Tumour weight (*n* = 6 per group). **c** Body weight (*n* = 6 per group). **d** Proliferation of MIAPaCa-2 cells in tumours. Tissue sections were stained for Ki67. The percentage of Ki67-positive cells (*n* = 14 and 15 for vehicle and AdipoRon group, respectively) is shown on the right. Nuclei were also stained with DAPI. Bars: 100 μm. **e** Tumour angiogenesis. Tissue sections were stained for CD31. Nuclei were also stained with DAPI. The vessel density (*n* = 10 and 14 for vehicle and AdipoRon group, respectively) is shown on the right. Bars: 100 μm. **f**, **g** Viability of patient-derived pancreatic cancer cells. Representative images of staining with FITC-labelled anti-EpCAM antibody and PI (**f**). White and yellow arrowheads represent EpCAM^−^PI^+^ and EpCAM^+^PI^+^ cells, respectively. Bars: 100 μm. The percentage of EpCAM^+^PI^+^ cells (**g**). Bar: 50 μm. **h** Effect of MitoTEMPO on AdipoRon-induced cell death of pancreatic cancer cells derived from patients. The cells were pre-treated with 10 μM MitoTEMPO for 1 h and then treated with 100 μM AdipoRon in the absence or presence of 10 μM MitoTEMPO for 40 h. For **a**–**e** and **h**, error bars represent standard deviation. *P < 0.05, **P < 0.01, ***P < 0.001 by Student’s *t*-test or ANOVA test

On the basis of the above observations, we investigated the anticancer effects of AdipoRon. To achieve this goal, we subcutaneously injected MIAPaCa-2 cells into nude mice. From day 12 after inoculation, the mice were orally administered 60 mg/kg AdipoRon every other day. We observed a significant retardation of tumour growth (Fig. 7a, b) without body weight loss (Fig. 7c). Ki67 staining of the sections prepared from AdipoRon-treated tumours revealed an inhibition of the proliferation of MIAPaCa-2 cells in treated compared with untreated tumours (Fig. 7d). In addition, we noticed a significant reduction of microvessel density, suggesting either indirect or direct inhibition of angiogenesis by AdipoRon (Fig. 7e).

### AdipoRon induces cell death of cancer cells derived from patients with pancreatic cancer

We tested the effect of AdipoRon on the survival of human pancreatic cancer cells prepared from surgically removed tissues. EpCAM staining revealed that both EpCAM^+^ and EpCAM^−^ cells were present in the dissociated pancreatic cancer cell clumps (Fig. S11), indicating that pancreatic cancer cells could be discriminated from stromal cells after staining for EpCAM. Next, we treated the dissociated cell preparations with AdipoRon followed by staining with PI and FITC-labelled EpCAM antibody to evaluate cancer cell death by counting PI^−^EpCAM^+^ and PI^+^EpCAM^+^ cells. The results showed that AdipoRon greatly increased the number of PI-positive cells (Fig. 7f, g). Notably, as observed for MIAPaCa-2 cells, AdipoRon-induced cell death of patient-derived pancreatic cancer cells was blocked by MitoTEMPO (Fig. 7h).

## Discussion

In the present study, we demonstrated that AdipoRon induced growth inhibition and cell death of pancreatic cancer cell lines. Unexpectedly, the death-inducing effect of AdipoRon was independent of AdipoRs. This finding was supported by the observations that APN slightly inhibited growth but did not induce cell death and that knockdown of AdipoRs was marginally protective against AdipoRon-induced cell death.

The type of AdipoRon-induced cell death of MIAPaCa-2 cells seems to be mainly RIPK1-dependent necroptosis-like cell death, as judged from prominent cytoplasmic swelling (ballooning), annexin V^+^/PI^+^ staining, LDH release, ATP depletion and the sensitivity to Nec-1. Because MIAPaCa-2 cells do not express RIPK3^31^, the cell death is not classical necroptosis mediated by a signalling complex composed of RIPK1, RIPK3 and mixed-lineage kinase domain-like (MLKL)^45^. Both caspase-independent apoptosis mediated by tAIF and autophagic cell death also seemed to take place in small fractions of AdipoRon-treated cells. Because it has been shown that apoptosis and necrosis can be induced simultaneously^46,47^ and that autophagy may be interconnected with necroptosis^48^, these cell death modalities would also be concurrently induced in AdipoRon-treated cells.

Mechanistically, we found that ERK1/2 phosphorylation is involved in AdipoRon-induced cell death. A rise in [Ca^2+^]_i_ could trigger ERK1/2 activation, which may be further augmented by activated ERK1/2. The ER calcium channel RyR was likely responsible for the rise in [Ca^2+^]_i_ because the RyR inhibitors, ruthenium red and dantrolene, partially ameliorated AdipoRon-induced cell death. However, since the amelioration by dantrolene was weak, we cannot exclude the possibility of other mechanisms. Furthermore, whether ERK1/2 activation is directly involved in the regulation of RyR function remains unknown. These issues await further investigation. Subsequent aberrant increase in [Ca^2+^]mt that is caused by opening of MCU was found to be critical for AdipoRon-induced cell death, as corroborated by observations that Ru360 and siRNA-mediated MCU knockdown attenuated cell death. The mechanism by which AdipoRon stimulates MCU opening also remains unresolved. ERK1/2 activation occurs upstream of MCU because U0126 inhibited [Ca^2+^]mt influx. It has been shown that dysregulation of [Ca^2+^]mt levels is closely associated with mtROS generation and cell death^40,49^. Increased Ca^2+^ activates calpain 1 (μ-calpain) in both cytosol and mitochondria and may cleave AIF^50^, leading to caspase-independent apoptosis. When Ca^2+^ is overloaded in the mitochondrial matrix, the ions interact with cyclophilin D to induce opening of the mitochondrial permeability transition pore (PTP) and stimulate the generation of mtROS^51^, which in turn promotes the opening of PTP^49^, leading to lipid peroxidation and a decrease in mitochondrial membrane potential. Damage to the mitochondrial membrane would decrease complex I activity, which also stimulates ROS production. Consistent with these observations, AdipoRon increased mtROS, which subsequently led to mitochondrial membrane lipid peroxidation, decreases in complex I activity and mitochondrial membrane potential, and activation of AIF. Because MitoTEMPO attenuated AdipoRon-induced cell death, the increases in mtROS production and lipid peroxidation participate in an essential priming step(s) of cell death (Fig. S12).

Insights into the signalling pathways underlying AdipoRon-induced cell death have revealed that AdipoRon activates both death and survival signalling pathways. As mentioned above, the death signalling pathways involve AdipoR-independent RIPK1/ERK1/2 activation that leads to mitochondrial dysfunction. The survival signalling pathways involve AdipoR-dependent activation of AMPK that may inhibit cell proliferation via mTOR and PPARα/γ activation that may participate in the expression of genes encoding antioxidant-detoxifying enzymes. AMPK was probably activated by an increase in AMP/ATP ratio. Inhibition of the survival signals by the AMPK and p38 MAPK inhibitors slightly but significantly enhanced AdipoRon-induced cell death. These results indicate that the survival signals can be active to resist AdipoRon-induced cell death. Nevertheless, AdipoRon eventually induced the death. This may be because AdipoRon rapidly dictates cell death and AdipoRon-evoked survival signals are weak and, as a result, the death signals may predominate over the survival signals (Fig. S12). However, we cannot explain why knockdown of AdipoRs, which should impair survival signals, did not enhance AdipoRon-induced cell death. It may be possible that some factors like off-target effects of siAdipoRs could affect the death. By contrast, APN activated only survival signals and did not activate, or rather inhibited, ERK1/2-mediated death signals (Fig. S12). These results coincide with a previous report showing that APN contributes to pancreatic cancer progression by conferring apoptosis resistance to pancreatic cancer cells^27^.

We demonstrated that oral administration of AdipoRon exhibited an anticancer effect against MIAPaCa-2 tumours. AdipoRon reduced the number of Ki67-positive cells in subcutaneous tumours. However, whether RIPK1-dependent and RIPK3-independent necroptosis was induced in AdipoRon-administered MIAPaCa-2 tumours remains unknown. It can also be argued that as AdipoRon is rapidly cleared from plasma, the antitumor effect would be indirect. However, after oral administration of 50 mg/kg AdipoRon into mice, the maximum concentration of AdipoRon in plasma was reported to be 11.8 µM^29^, which is within the effective concentrations for suppressing the growth of MIAPaCa-2 cells (Fig. 1d). In addition, after lipid peroxidation is initiated by AdipoRon-induced mtROS, free radical lipid peroxidation may proceed via a chain reaction mechanism through the polyunsaturated fatty acids of phospholipids in the mitochondrial membrane, ultimately leading to cell death^52^. Therefore, we believe that administration of 60 mg/kg AdipoRon may be able to induce necroptosis in MIAPaCa-2 tumours. Moreover, AdipoRon inhibited tumour angiogenesis. A previous study has shown that activation of AMPK in endothelial cells inhibits tube formation induced by bone morphogenetic protein 9^53^, suggesting that AdipoRon may inhibit angiogenesis by activating AMPK in endothelial cells. AdipoRon may also suppress the infiltration of tumour-associated macrophages that are known to facilitate angiogenesis^54^. Combined with these effects, AdipoRon may be able to suppress tumour growth.

Since AdipoRon was identified as an AdipoR agonist^29^, the cell killing effect was unexpected. Nonetheless, the observations that AdipoRon retarded tumour growth by oral administration and killed primary human pancreatic cancer cells suggest that AdipoRon is a valuable therapeutic agent. It has been suggested that conditions associated with diabetes promote pancreatic carcinogenesis and that pancreatic cancer causes the associated diabetes^55^. Therefore, AdipoRon could also be used not only to prevent pancreatic carcinogenesis associated with diabetes but also to treat pancreatic cancer-associated diabetes. Further studies aimed at application of AdipoRon to the treatment of pancreatic cancer are warranted.

## Materials and methods

### Cells and cell culture

The human pancreatic cancer cells, AsPC-1, BxPC-3, CAPAN2, CFPAC, HPAF, MIAPaCa-2, Panc-1 and SW1990, were used in this study^56^. MIAPaCa-2 and Panc-1 cells were obtained from the RIKEN BRC Cell Bank (Tsukuba, Japan), and other human pancreatic cell lines were purchased from the ATCC (Manassas, VA, USA). These cells were cultured in Dulbecco’s Modified Eagle’s Medium (DMEM) containing 10% FBS and 40 μg/ml gentamicin. Human pulmonary alveolar epithelial cells (HPAEpiC) were purchased from ScienCell (Carlsbad, CA, USA) and cultured in alveolar epithelial cell medium (AEpiCM) supplemented with 2% foetal bovine serum (FBS), epithelial cell growth supplement (EpiCGS) and penicillin/streptomycin. All cells were maintained in a humidified atmosphere with 21% O_2_/5% CO_2_. All cell lines were free of mycoplasma contamination as evaluated using the e-Myco Mycoplasma PCR Detection Kit (Cosmo Bio Co Ltd., Tokyo, Japan).

### Reagents

AdipoRon was obtained from AdipoGen (San Diego, CA, USA), and human recombinant HMW-rich APN was from BioVender (Brno, Czech Republic). AdipoRon was freshly prepared in dimethyl sulfoxide (DMSO) before use. MitoTEMPO, chloroquine (CQ), ruthenium red and KN-93 were purchased from Sigma-Aldrich Japan (Tokyo, Japan) and Z-VAD-fmk was from Peptide Institute, Inc. (Osaka, Japan). Necrostatin-1 (Nec-1) and necrostatin-1s (Nec-1s) were provided by BioVision, Inc. (Milpitas, CA, USA). BAPTA-AM was supplied by AdooQ Bioscience LLC (Irvine, CA, USA), and SB203580, BML-275 and Akt inhibitor were from Calbiochem (San Diego, CA, USA).

### Cell cycle analysis

MIAPaCa-2 cells treated with solvent (DMSO) alone or AdipoRon were fixed in 70% ethanol and stored at −20 °C until use. The fixed cells were washed with Dulbecco’s PBS (DPBS) and incubated with 100 µg/ml RNase A and 50 µg/ml PI (Sigma-Aldrich Japan). The cells were then subjected to flow cytometric analysis using a FACSCalibur flow cytometer (BD Biosciences, Franklin Lakes, NJ, USA)^56^.

### Cell growth and viability assay

Cell growth and viability were measured using the MTT (3-(4,5-dimethylthiazol-2-yl)-2,5-diphenyltetrazolium bromide) assay. Briefly, cells (1 × 10^4^ cells/well) were cultured in 96-well tissue culture plates and pre-treated in triplicate in 100 µl medium with or without different concentrations of inhibitors for 1 h, followed by solvent alone, AdipoRon or APN for the indicated period. At the end of the incubation, 10 µl of MTT (1 mg/ml) (Sigma-Aldrich Japan) was added to the wells to allow the formation of MTT formazan crystals for 4 h. After the medium was removed, the crystals were solubilized in 100 µl of DMSO. The absorbance was recorded at 550 nm^56^.

### LDH release

LDH release was assayed using the Cytotoxicity LDH Assay Kit (Dojindo, Kumamoto, Japan) according to the manufacturer’s protocol.

### Measurement of caspase-3/9 activity

Caspase-3 or caspase-9 activity was measured with the APOPCYTE Caspase-3 or Caspase-9 Colorimetric Assay Kit (MBL, Nagoya, Japan), respectively, according to the manufacturer’s protocol.

### Annexin V/propidium iodide (PI) staining

The Annexin V-FITC Apoptosis Detection Kit (Beckman Coulter, Inc., Pasadena, CA) was used to detect annexin V and/or PI-positive cells. Briefly, MIAPaCa-2 cells were stained with Annexin V-FITC for 15 min at room temperature in the dark and PI in ice-cold Binding Buffer. Annexin V and/or PI-positive cells were analysed using a FACSCalibur flow cytometer^56^.

### AdipoR knockdown by small interference (si) RNAs

For the transient knockdown of AdipoR1 and AdipoR2, MIAPaCa-2 cells were transfected with 20 nM AdipoR1 (siR1) or AdipoR2 siRNA (siR2) (Santa Cruz Biotechnology, Santa Cruz, CA, USA) with Lipofectamine RNAiMAX reagent (Thermo Fisher Scientific) according to the manufacturer’s protocol. Silencer Negative Control #1 siRNA (Ambion, Thermo Fisher Scientific, Waltham, MA, USA) was used as a control. Two days after transfection, the cells were subjected to total RNA isolation and viability assays.

### Quantitative RT-PCR

Quantitative RT-PCR (qRT-PCR) was performed on cDNA using THUNDERBIRD SYBR qPCR Mix (TOYOBO, Osaka, Japan) and 0.3 μM primers in a 20 μl volume. The reactions were run on a Thermal Cycler Dice Real Time System TP860 (TaKaRa, Shiga, Japan). The PCR protocol consisted of an initial denaturation step at 95 °C for 1 min and 40 cycles of denaturation (95 °C for 15 s) and extension (60 °C for 1 min). Dissociation curve analyses were performed to confirm the PCR product identity and to differentiate specific amplification from non-specific products by denaturation (95 °C for 15 s), annealing (60 °C for 30 s), and slow heating to 95 °C. The mRNA expression level of each gene was normalized to GAPDH. The specific primer sets are shown in Supplemental Table SI.

### Preparation of cell extracts and western blotting

Cells were lysed in RIPA buffer (50 mM Tris-HCl, pH 7.4, 150 mM NaCl, 1% NP-40, 0.5% deoxycholate, 0.1% sodium dodecyl sulphate, 2 mM EDTA, protease inhibitor cocktail and phosphatase inhibitor cocktail) on ice for 20 min. The lysates were centrifuged at 12,000×*g* for 10 min at 4 °C, and the supernatants were used for western blot analyses. The primary antibodies used were anti-BAD, anti-Bax, anti-Bcl-2, anti-Mcl-1, anti-Bcl-XL, anti-p27, anti-AIF, anti-LC3B, anti-AMPK (CST), anti-phospho-AMPK (CST), anti-p38 MAPK (CST), anti-phospho-p38 MAPK (CST), anti-ERK, anti-phospho-ERK, anti-ACC1 (CST) and anti-phospho-ACC1 (CST). These primary antibodies were used at a 1:1000 dilution. The secondary antibodies were HRP-conjugated rabbit or anti-mouse IgG (1:3000 dilution, CST). For loading controls, anti-β-actin antibody was used. Signals were visualized using ECL plus (GE Healthcare, Little Chalfont, UK). The membranes were scanned with a Luminoimaging Analyzer LAS4000 (GE Healthcare).

### Immunofluorescent staining

MIAPaCa-2 cells treated with solvent alone or AdipoRon were fixed with 4% formaldehyde/5% sucrose in DPBS for 20 min, rinsed with DPBS and permeabilized with 0.5% Triton X-100 in DPBS for 4 min. The cells were blocked with 3% BSA/0.1% glycine in DPBS for 1 h, rinsed, and then incubated with rabbit polyclonal anti-LC3B antibody (1:200 dilution) for 1 h. After extensive washing with DPBS, the cells were incubated with Alexa Fluor 488-conjugated goat anti-rabbit IgG (1:300 dilution, Invitrogen) for 1 h. The cells were counterstained with DAPI and observed under a laser scanning confocal microscope (Fluoview FV1000, Olympus, Tokyo, Japan).

### Immunohistochemistry

MIAPaCa-2 tumour tissues were surgically removed and immediately embedded and frozen in OCT compound. Cryostat sections (8 μm thick) were fixed in 4% paraformaldehyde for 10 min, blocked with 1% BSA in DPBS and then incubated with anti-Ki67 antibody (1:200 dilution, Novus Biologicals, Littleton, CO, USA) or rat anti-mouse monoclonal CD31 antibody (1:100 dilution, BD Biosciences, 550274). After extensive washing with DPBS, the sections were incubated with Alexa Fluor 594-conjugated goat anti-rat IgG (1:300 dilution, Invitrogen) for 1 h, counterstained with 1 µg/ml DAPI and observed under a confocal laser scanning microscope (Fluoview, Olympus). To determine the vessel density, pixel values of the CD31-positive areas were calculated for each image to determine the tumour vessel density using ImageJ software (National Institutes of Health).

### Measurement of mitochondrial Ca^2+^ levels with Rhod2-AM

MIAPaCa-2 cells incubated with solvent alone or 100 µM AdipoRon or APN for various times were loaded with 10 µM Rhod2-AM (Dojindo) for 15 min. Rhod2-AM has a net positive charge, facilitating its sequestration into mitochondria through membrane potential-driven uptake. The AM ester of the probe is rapidly cleaved in the mitochondria to yield the Rhod2 indicator, which displays a large increase in fluorescence intensity upon binding to Ca^2+^^37^. After loading Rhod2-AM, the cells were immediately subjected to flow cytometry analysis or observed under a confocal laser scanning microscope.

### Measurement of intracellular Ca^2+^ level with Fluo4

MIAPaCa-2 cells were seeded into a 96-well fluorescent plate (15,000 cells per well) and cultured overnight. The cell culture medium was replaced with recording medium (20 mM HEPES, 115 mM NaCl, 5.4 mM KCl, 1.8 mM CaCl_2_, 0.8 mM MgCl_2_ and 13.8 mM glucose, pH 7.4) containing 3 µM Fluo4-AM (Dojindo) (100 µl per well). After a 1-h incubation, the cells were washed three times with DPBS, and pre-warmed recording medium (100 µl per well) was added. Baseline recordings were acquired for 5 min in a DTX-880 multimode plate reader (Beckman-Coulter, Brea, CA, USA), followed by the addition of vehicle or AdipoRon. Fluorescent signals were then recorded every 30 s for at least an additional 5 min from a population of cells in a central region of each well at an excitation wavelength of 485 nm and emission wavelength of 535 nm.

### Measurement of ROS generation

ROS production was monitored by flow cytometry or under a confocal laser microscope with 2′,7′-dichlorodihydrofluoresceindiacetate (H_2_DCF-DA) (Molecular Probe-Life Technologies, Carlsbad, CA, USA) and the mitochondrial superoxide indicator MitoSOX Red (Invitrogen) as probes. Briefly, MIAPaCa-2 treated with solvent alone, AdipoRon or APN for various times were incubated with 10 µM H_2_DCF-DA or 5 µM MitoSOX Red in serum-free DMEM for 10 min. For flow cytometry analysis, the medium was removed, and the cells were detached with a brief treatment of 0.25% trypsin in Hank’s balanced salt solution. After addition of fresh culture medium, the cells were collected by centrifugation, washed once with DPBS and suspended in DPBS.

### Analysis of mitochondrial lipid peroxidation

Lipid peroxides in mitochondria were detected using a cell-permeable fluorescent probe, MitoPeDPP (3-[4-(perylenylphenylphosphino)phenoxy]propyltriphenylphosphonium iodide) (Dojindo), which specifically localizes in mitochondria due to the triphenylphosphonium moiety and can be applied for lipophilic peroxide imaging^57^. Briefly, MIAPaCa-2 cells treated with solvent alone or 100 µM AdipoRon for up to 3 h were washed twice with DPBS and incubated with 0.5 µM MitoPeDPP and 100 nM MitoTracker Red CMXRos (Thermo Fisher Scientific) for 15 min. After rinsing with DPBS, the cells were observed under a confocal laser microscope.

### Measurement of mitochondrial membrane potential

The mitochondrial membrane potential was monitored by staining the cells with 100 nM MitoTracker Red CMXRos for 10 min. The stained cells were observed under a laser confocal microscope.

### Mitochondrial complex I assay

Complex I activity was assayed using a Complex I Enzyme Activity Microplate Assay Kit (Abcam) according to the manufacturer’s protocol.

### ATP quantification

The intracellular ATP level was quantified using “Cell” ATP Assay reagent (TOYO B-Net Co., LTD. Osaka).

### Fatty acid oxidation (β-oxidation) assay

Fatty acid oxidation was measured in triplicate by quantitating the production of ^3^H_2_O from [9,10-^3^H]palmitate as described previously^58^. Briefly, 200 µl per well of PBS containing 1 µCi [9,10-^3^H] palmitate (30 Ci/mmol, PerkinElmer Life Science Products) bound to fatty acid-free albumin was added to MIAPaCa-2 cells in 24-well microplates. Incubation was carried out for 2 h at 37 °C. In some experiments, 25 µM etomoxir or 500 µM l-carnitine was added to the incubation medium. After incubation, the mixture was removed and added to 200 µl of cold 10% trichloroacetic acid. The tubes were centrifuged for 10 min at 2200×*g* at 4 °C, and aliquots of supernatants (350 µl) were removed, mixed with 55 µl of 6 N NaOH, and applied to ion-exchange resin (Dowex 1×2-400 resin, Sigma-Aldrich). The columns were washed twice with 750 µl of water, and the eluates were counted. Palmitate oxidation rates were expressed as ^3^H_2_O counts/10^6^ cells/h.

### Animal experiments

All animal experiments were performed in compliance with the institutional guidelines for the care and use of animal research. The protocol was approved by the IZUMO Campus Animal Care and Use Committee of Shimane University (Permission Number: IZ27-37). MIAPaCa-2 cells (1 × 10^6^ cells/mouse) were subcutaneously implanted with 50% Matrigel into 5-week-old-female nude mice (BALB/c nu/nu, Japan SLC, Shizuoka, Japan). Twelve days after injection, the mice were randomly allocated into two groups (Control and AdipoRon groups) of 6 mice per group. In the AdipoRon group, mice were orally administered 60 mg/kg AdipoRon using gastric sonde every other day. In the Control group, mice were administered solvent alone in DPBS. The volumes of MIAPaCa-2 tumours were evaluated by measuring two perpendicular diameters with callipers. The tumour volume (*V*) was calculated using the following equation: *V* = (*a*^2^ × *b*)/2, where *a* is the small diameter and *b* is the large diameter.

### Preparation of cell suspensions of pancreatic cancer tissues

The use of the cancer tissues that were surgically excised from patients with pancreatic cancer was approved and reviewed by the Ethics Committee of Shimane University Hospital (Approval no. 1348) in accordance with the Helsinki declaration. The patients provided consent for their pancreatic cancer specimens to be used in the future for the purpose of evaluation of diagnostic tests and anticancer effect of new potential drugs and molecular biological analyses. Informed consent was obtained from all patients. Cancer tissues were dissociated by first cutting them into small pieces with scissors and then treating them with 0.28 U/ml Liberase DH (Roche Life Science) at 37 °C for 2 h. After centrifugation at 200×*g* at 4 °C for 5 min, the cell pellet was washed with Hank’s balanced salt solution (HBSS) and passed through a Cell Strainer (100 μm). The cells and cell clumps that passed through were collected, washed with HBSS, suspended in fresh culture medium, and then seeded equally into the wells of 96-multiwell culture dishes. After a 24-h incubation, the cells were treated with AdipoRon.

### Statistics

All data are presented as the mean ± SD. No statistical method was used to predetermine the sample size. The researchers were not blinded to allocation during experiments and outcome assessment. Statistical significance between data sets was tested using the two-tailed Student’s *t*-test with an unpaired analysis. One-way ANOVA was used for multi-group data comparisons. To analyse data affected by two factors, two-way ANOVA was applied. *P* *<* 0.05 was considered significant.

## Electronic supplementary material

Supplemental material

Table S1
