# Supplementary material for: Antidiabetic adiponectin receptor agonist AdipoRon suppresses tumour growth of pancreatic cancer by inducing RIPK1/ERK-dependent necroptosis
Source: Cell Death Dis. 2018 Jul 23;9(8):804. doi: 10.1038/s41419-018-0851-z (PMC6056513; doi:10.1038/s41419-018-0851-z)
Supplement: Supplementary file 1 — Supplemental material [file 41419_2018_851_MOESM1_ESM.pdf]

Supplementary data

Antidiabetic adiponectin receptor agonist AdipoRon suppresses tumor growth of pancreatic cancer

Miho Akimoto,[1*] Riruke Maruyama,[2] Yasunari Kawabata,[3] Yoshitsugu Tajima,[3] and Keizo Takenaga[1]**

[1]Department of Life Science
[2]Department of Pathology
[3]Department of Digestive and General Surgery
Shimane University Faculty of Medicine, 89-1 Ennya, Izumo, Shimane 693-8501, Japan

*Present address: Department of Biochemistry, Teikyo University School of Medicine,
2–11-1 Kaga, Itabashi-ku, Tokyo 173–8605, Japan

**Correspondence: K. Takenaga, Present address: 666-2 Nitona, Chiba 260-8717, Japan. e-mail: ktakenaga@chiba-cc.jp

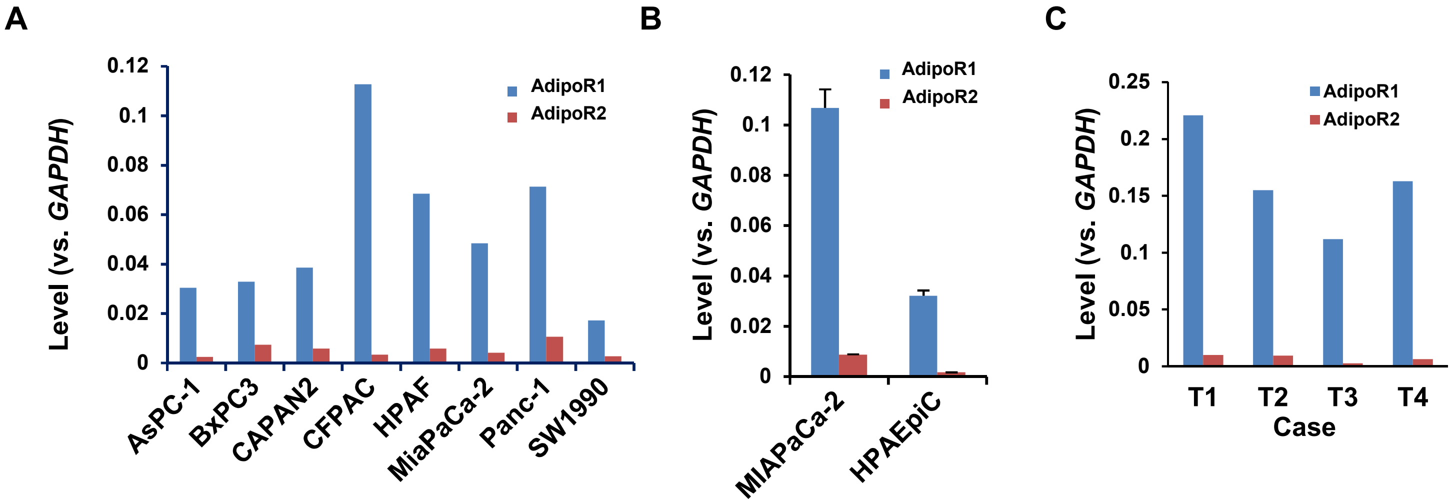

Fig. S1. Expression of AdipoR1 and AdipoR2. (A) Pancreatic cancer cell lines. (B) MIAPaCa-2 and normal pulmonary alveolar epithelial HPAEpiC cells. (C) Patient-derived pancreatic cancer tissues.

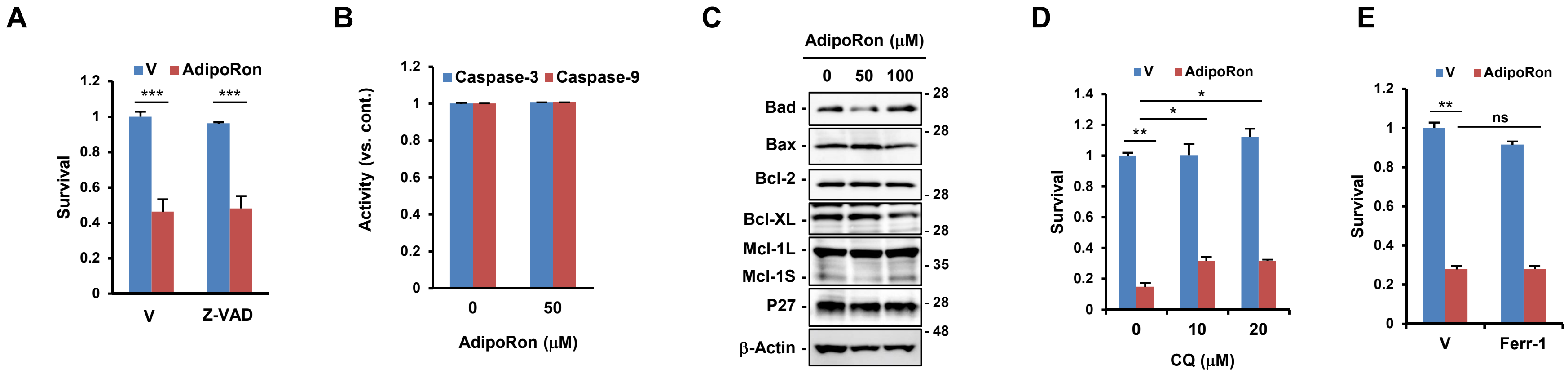

Fig. S2. Analyses of the type of death induced by AdipoRon in MIAPaCa-2 cells. (**A**) Effect of Z-VAD-fmk (Z-VAD) on AdipoRon-induced cell death. The cells were treated with vehicle alone (V) or 100 μM AdipoRon in the presence or absence of Z-VAD-fmk for 20 h. (**B**) Activities of caspase 3 and caspase 9 in AdipoRon-treated cells. The cells were treated with vehicle alone (V) or 50 μM AdipoRon for 24 h. (**C**) Expression of apoptosis-related proteins in AdipoRon-treated cells. The cells were treated with vehicle alone (V) or 50-100 μM AdipoRon for 40 h. Full size images of the Western blots presented are shown in Figure S13. (**D**) Effect of chloroquine on AdipoRon-induced cell death. The cells were treated with vehicle alone (V) or 100 μM AdipoRon in the presence or absence of the indicated concentrations of chloroquine (CQ) for 20 h. (**E**) Effect of ferrostatin-1 on AdipoRon-induced cell death. The cells were treated with vehicle alone (V) or 100 μM AdipoRon in the presence or absence of 50 μg/ml ferrostatin-1 (Ferr-1) for 20 h. *P<0.01, **P<0.001. ns, not significant.

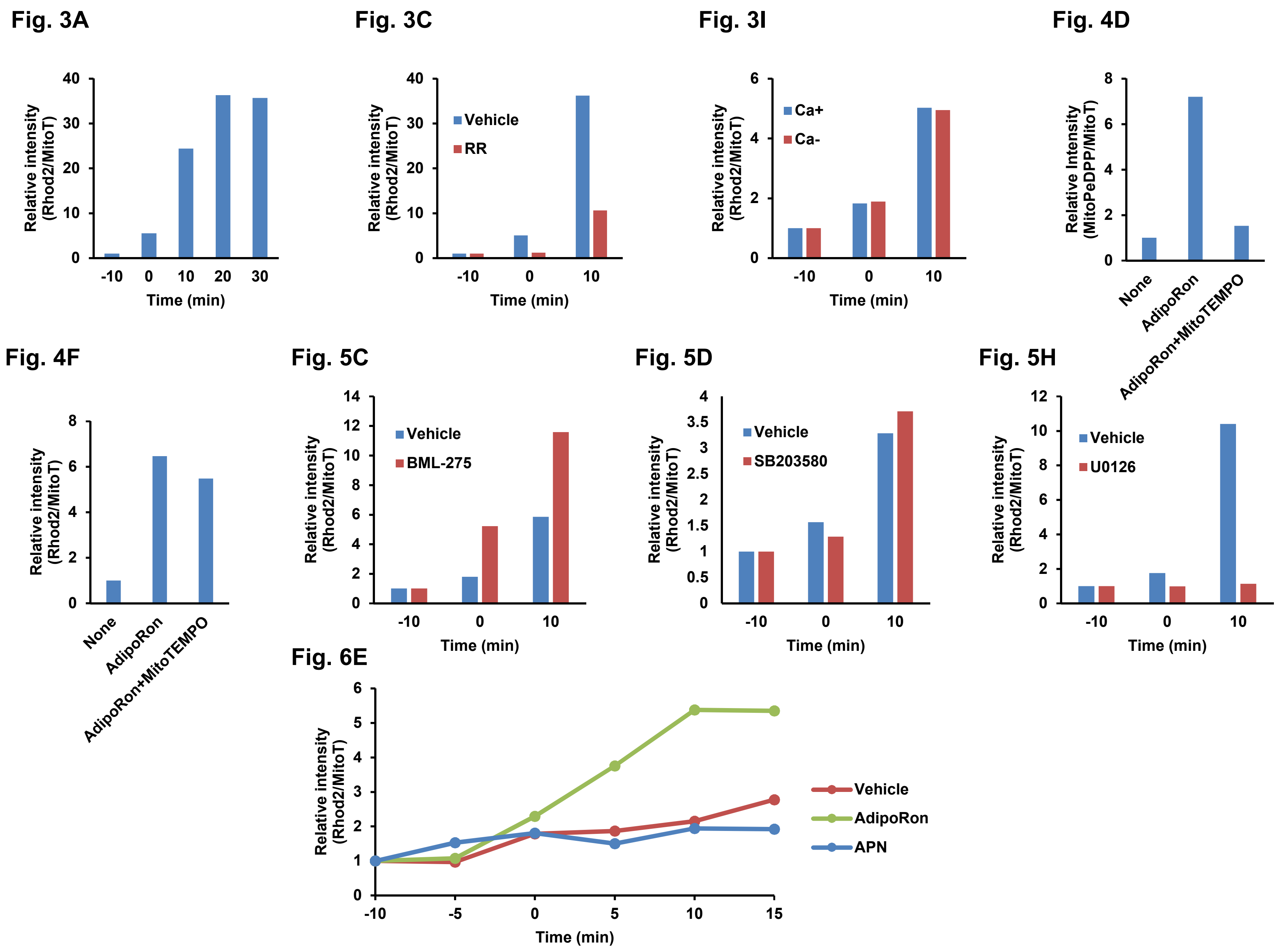

Fig. S3. **Semi-quantification of relative fluorescence intensity.** The pixel value of each fluorescence was calculated for each cell to determine the relative fluorescence intensity using the ImageJ software.

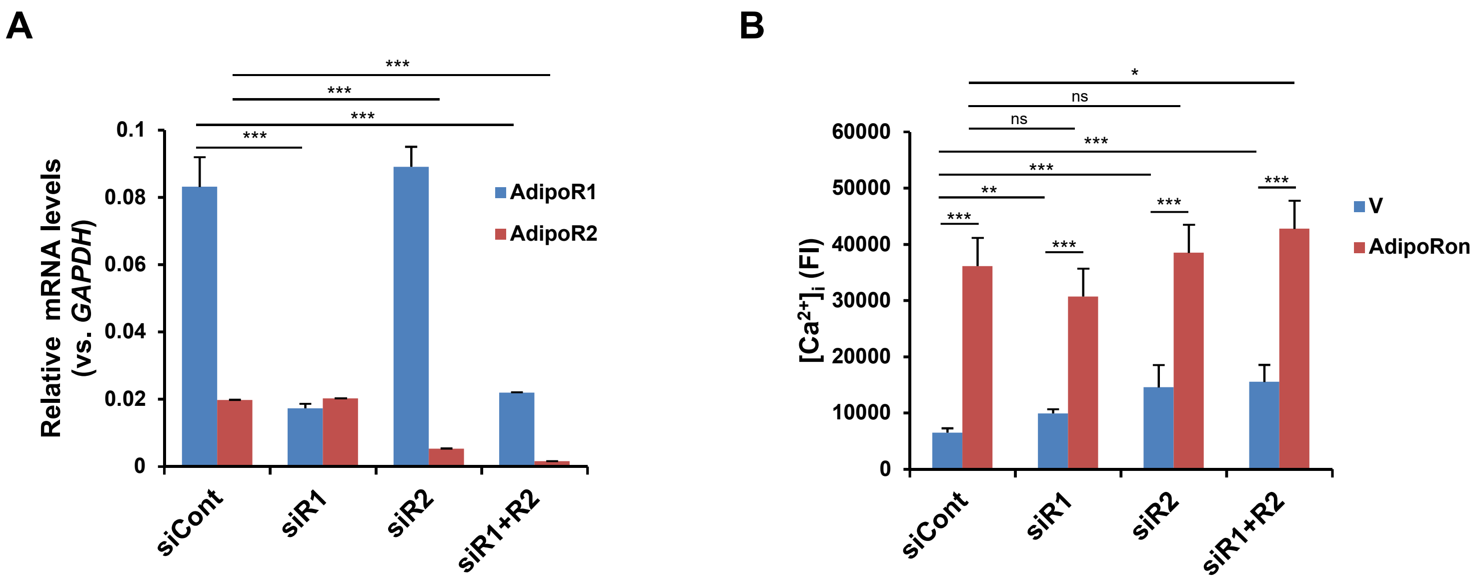

Fig. S4. Effect of knockdown of AdipoR1 and AdipoR2 on AdipoRon-induced $[Ca^{2+}]_i$ levels. (A) qRT-PCR analysis. MIAPaCa-2 cells were transfected with siAdipoR1 (siR1) or siAdipoR2 (siR2) or both. After 48 h, the mRNA expression of AdipoR1 and AdipoR2 was measured. (B) $[Ca^{2+}]_i$ levels. MIAPaCa-2 cells transfected with siAdipoR1 (siR1) or siAdipoR2 (siR2) or both were loaded with Fluo4-AM and then treated with solvent alone (V) or 100 μM AdipoRon for 30 min. FI: fluorescence intensity. *$P<0.05$, **$P<0.01$, ***$P<0.001$. ns, not significant.

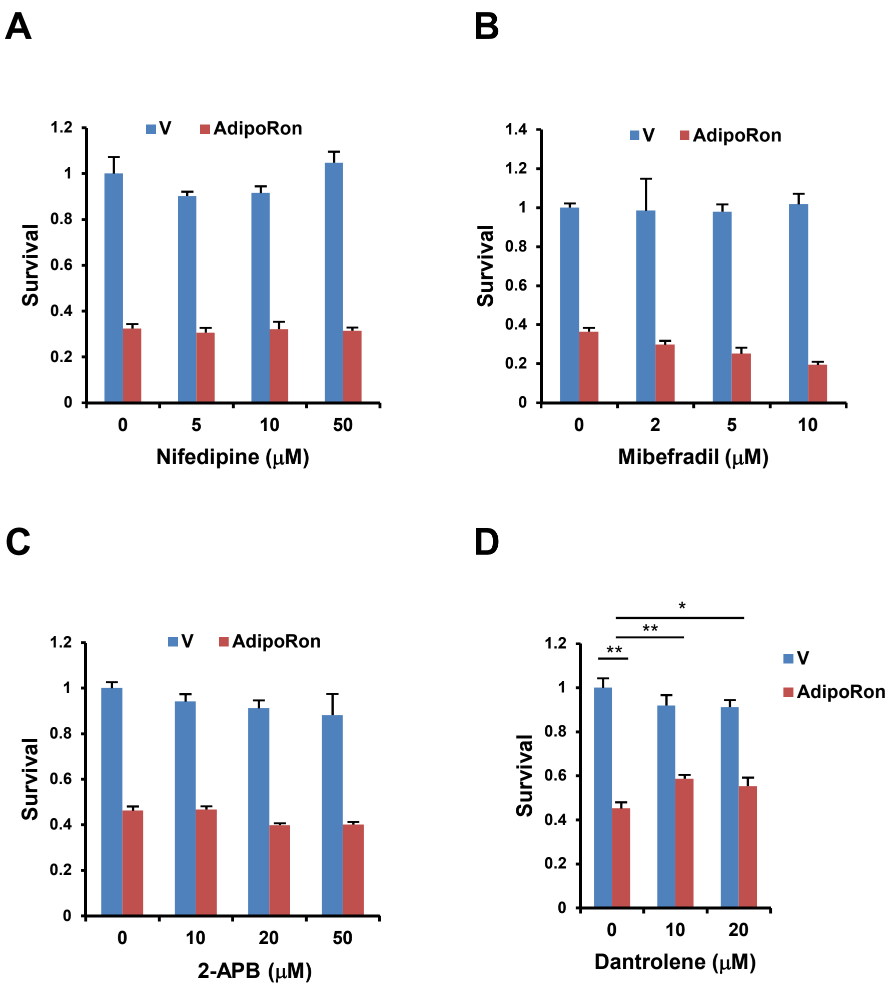

Fig. S5. Effect of calcium channel blockers on AdipoRon-induced cell death. The cells were pretreated with various concentrations of Nifedipine (A), mibefradil (B), 2-APB (C) or dantrolene (D) for 1 h and then with vehicle alone (V) or 100 μM AdipoRon for 24 h in the presence of inhibitors.

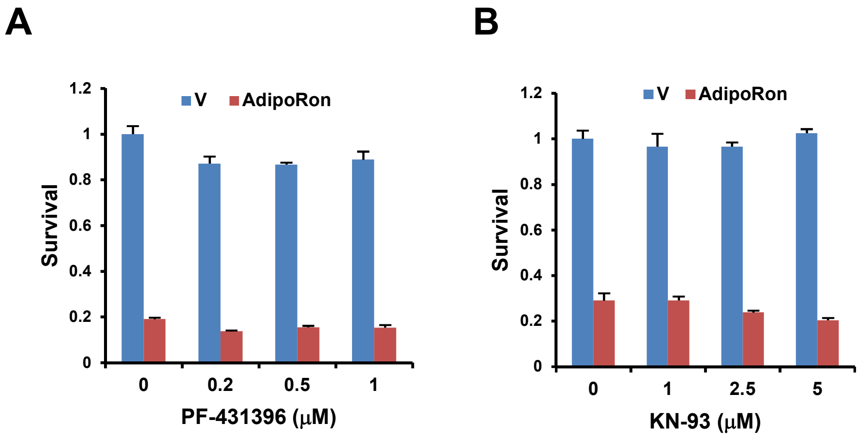

Fig. S6. Effect of PF-431396 and KN-93 on AdipoRon-induced cell death. The cells were pretreated with various concentrations of PF-431396 (A) or KN-93 (B) for 1 h and then with vehicle alone (V) or 100 μM AdipoRon for 20 h in the presence of inhibitors.

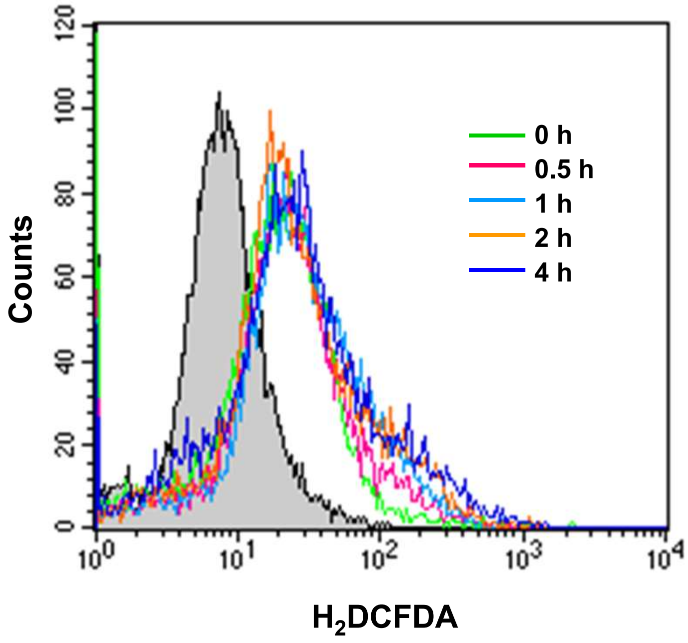

Fig. S7. Effect of AdipoRon on intracellular ROS production. The cells were treated with 100 μM AdipoRon for the indicated times. Intracellular ROS was examined by FACS after staining the cells with H_2DCF-DA.

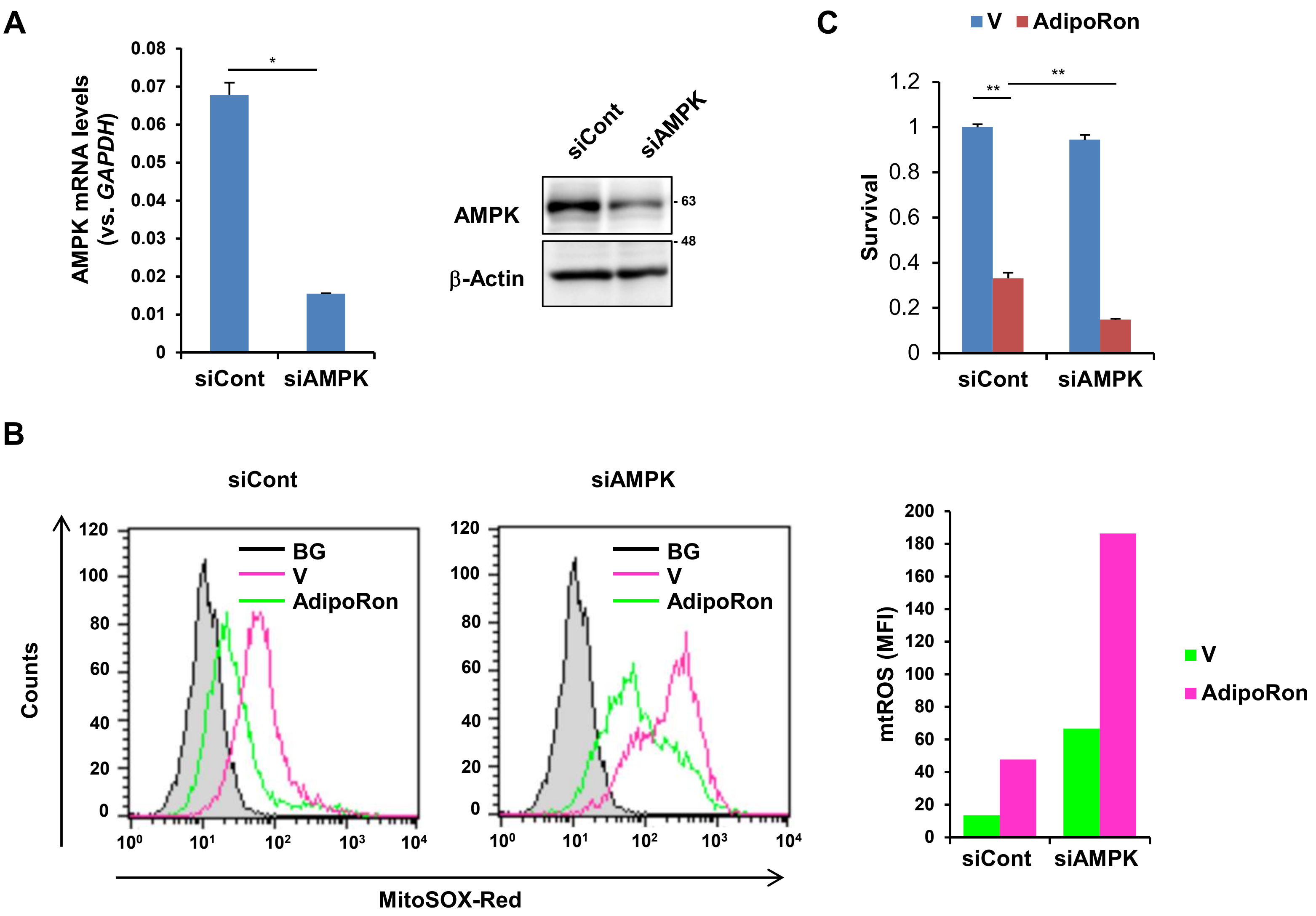

Fig. S8. Effects of knockdown of AMPK. (A) qRT-PCR analysis. MIAPaCa-2 cells were transfected with control siRNA (siCont) or AMPK siRNA (siAMPK). After 2 days, the expression of AMPK mRNA (left) and protein (right) were examined. Full size images of the Western blots presented are shown in Figure S13. (B) mtROS levels. Cells transfected with siCont or siAMPK were treated with 100 μM AdipoRon for 6 h. (C) Cell survival. Cells transfected with siCont or siAMPK were treated with 100 μM AdipoRon for 20 h.

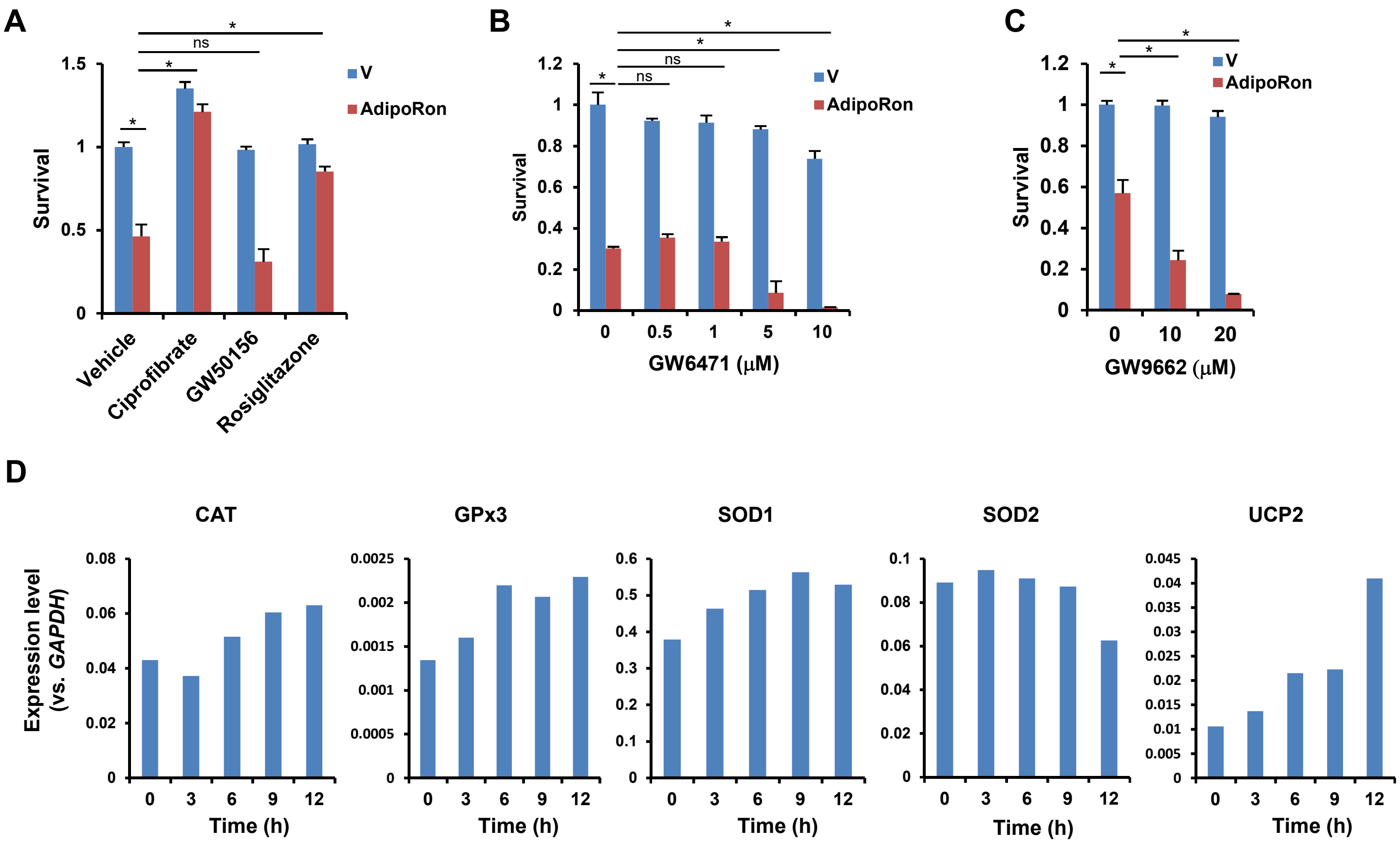

Fig. S9. Effects of PPAR agonists and antagonists on AdipoRon-induced cell death. MIAPaCa-2 cells were treated with vehicle alone (V) or 100 μM AdipoRon in the presence or absence of the indicated concentrations of drugs. (**A**) Effects of the PPARα agonist ciprofibrate (10 μM), the PPARβ agonist GW50156 (500 nM) and the PPARγ agonist rosiglitazone (10 μM). (**B**) Effect of the PPARα antagonist GW6471. (**C**) Effect of the PPARγ antagonist GW9662. (**D**) qRT-PCR analysis of the expression of antioxidant enzymes. MIAPaCa-2 cells were treated with 100 μM AdipoRon for the indicated time periods. CAT: catalase, GPx3: glutathione peroxidase 3, SOD1: superoxide dismutase 1, SOD2: superoxide dismutase 2, UCP2: uncoupling protein 2. *P<0.001.

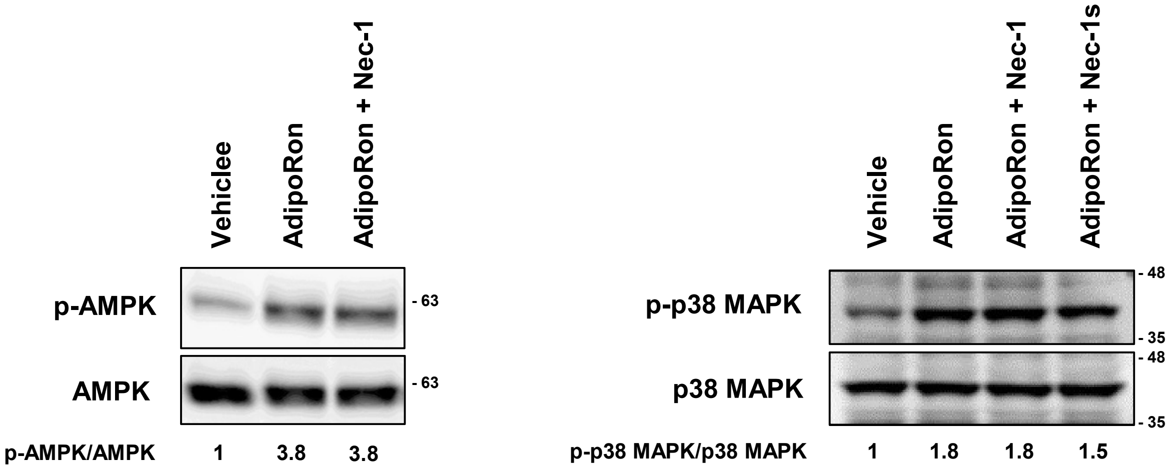

Fig. S10. Effect of Nec-1 and Nec-1s on AdipoRon-induced phosphorylation of AMPK and p38 MAPK. The cells were pretreated with 50 μM Nec-1 and 50 μM Nec-1s for 1 h and then with vehicle alone or 100 μM AdipoRon in the presence or absence of inhibitors for 30 min. The ratios of p-AMPK to AMPK and p-p38 MAPK to p38 MAPK are shown below. Full size images of the Western blots presented are shown in Figure S13.

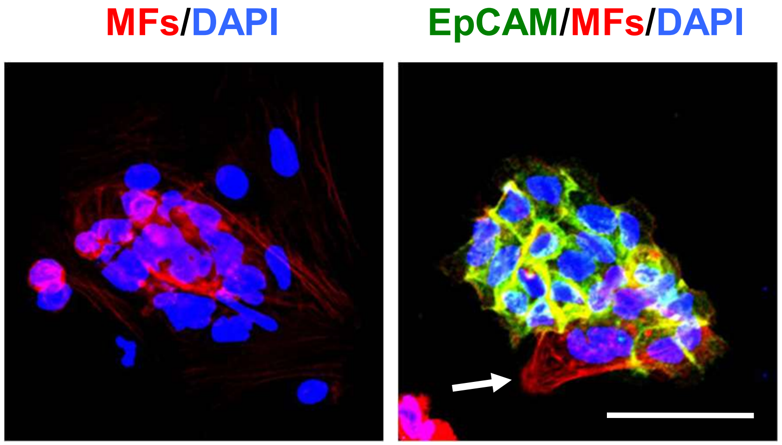

Fig. S11. Immunofluorescent staining of EpCAM of cell clumps isolated from pancreatic cancer tissues. Pancreatic cancer cells isolated from clinical specimen were fixed and immunostained with FITC-labeled anti-EpCAM antibody. The cells were also stained for microfilaments (MFs) with Atto 647N-Phalloidin and DAPI. The arrow indicates EpCAM-negative stromal cell. Bar: 50 μm.

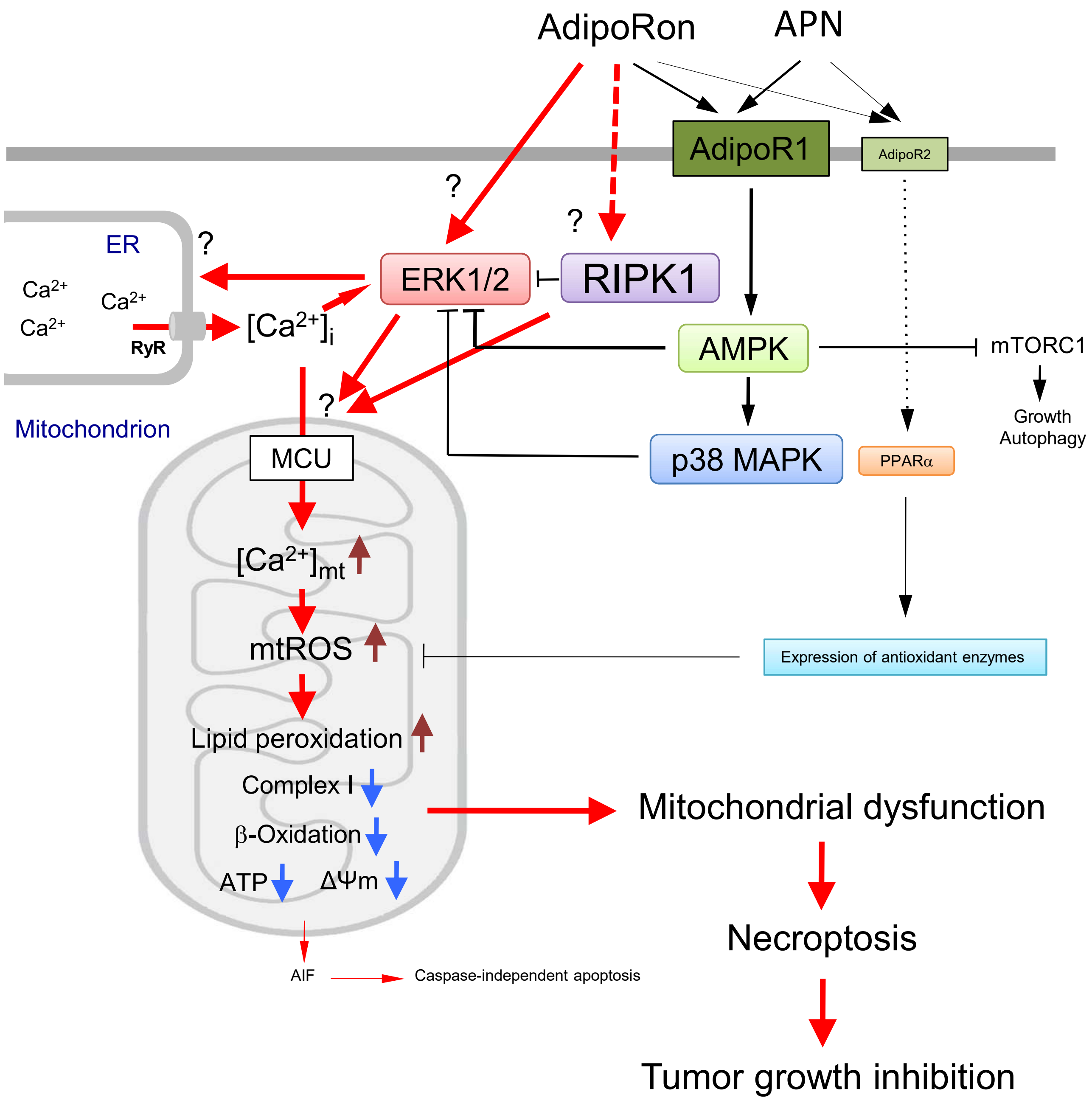

Fig. S12. Summary of the present findings.

Fig. 2F

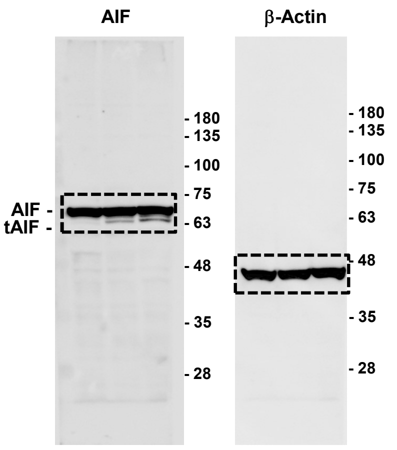

Fig. 2G

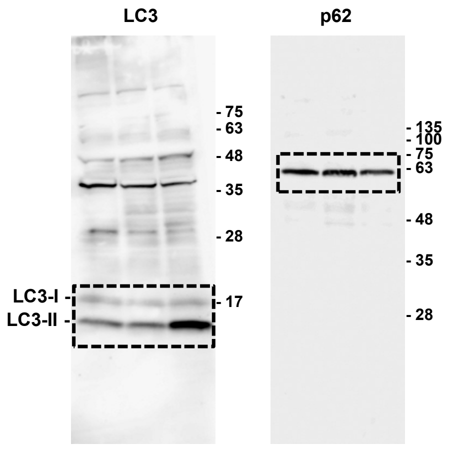

Fig. 5A

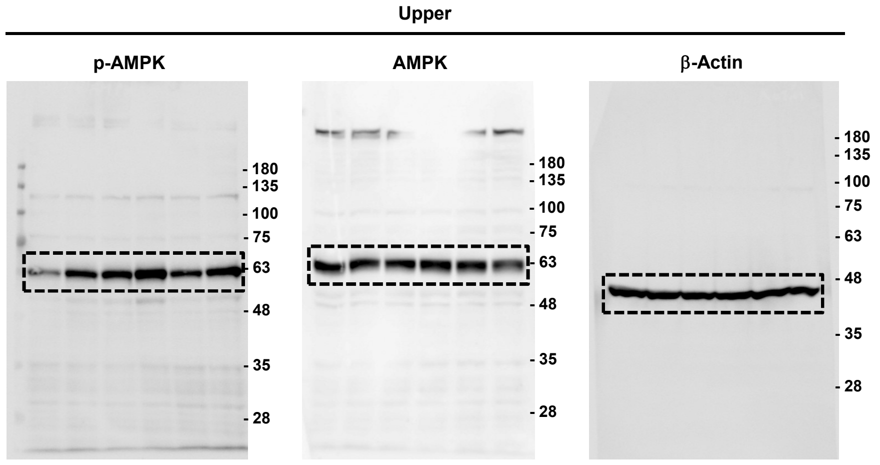

Fig. 3F

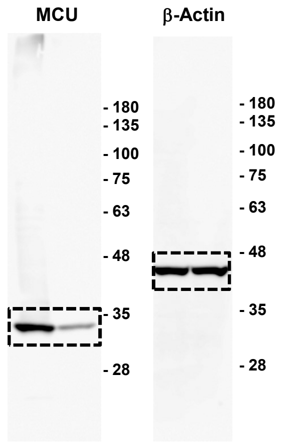

Fig. 5A

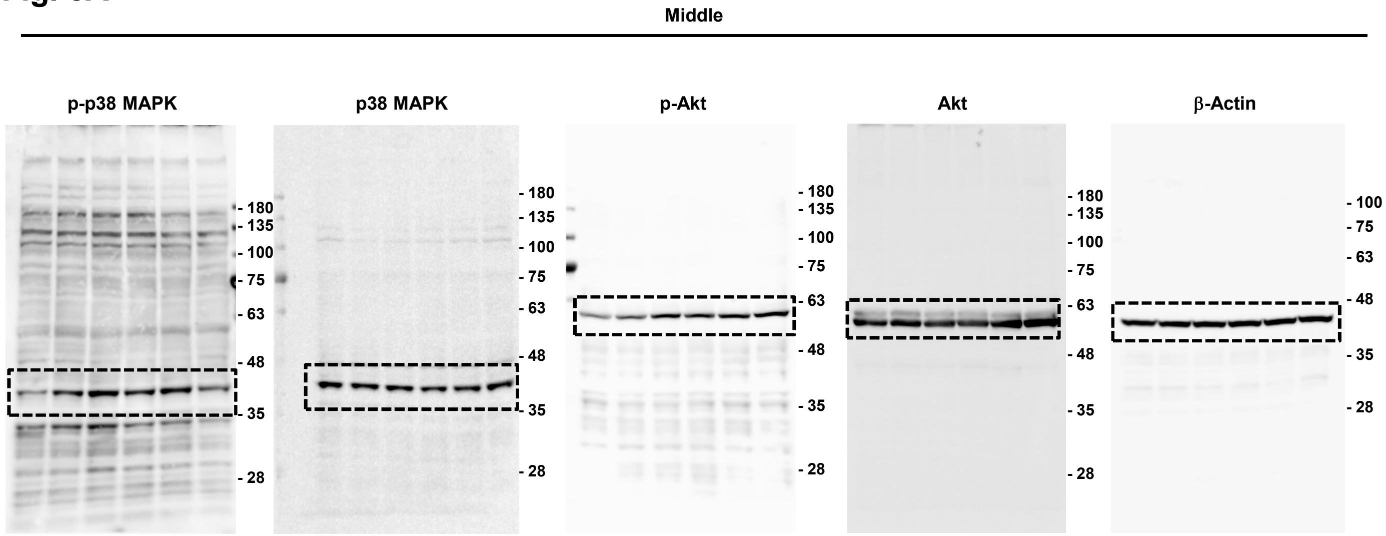

Fig. 5A

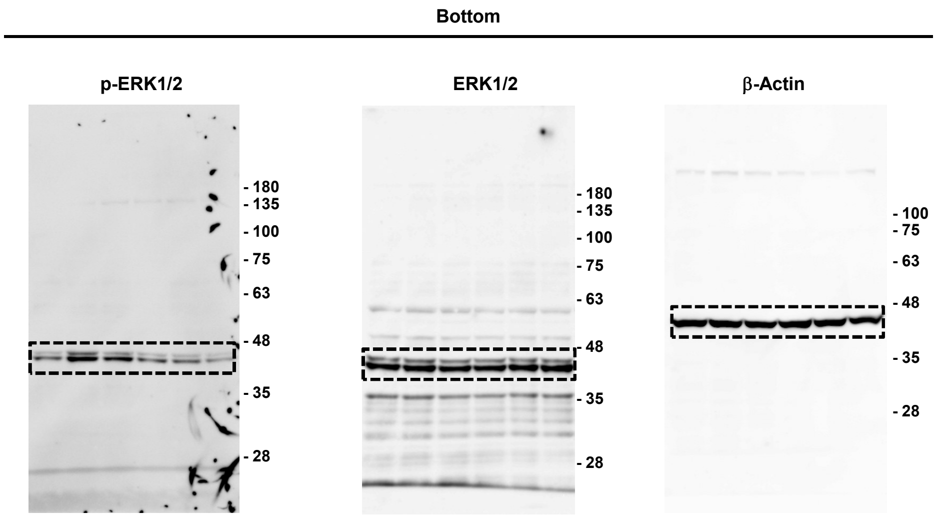

Fig. 5E

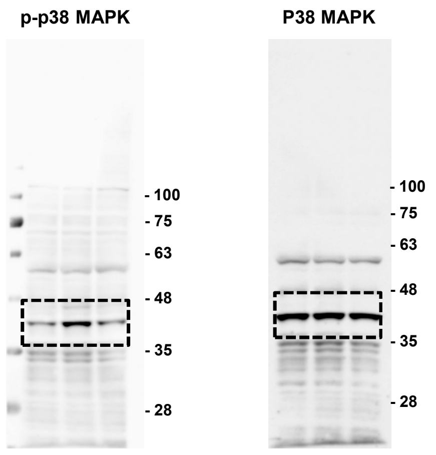

Fig. 5I

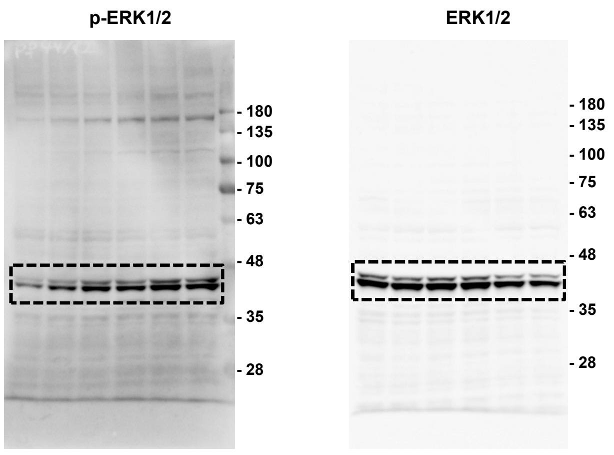

Fig. 6A

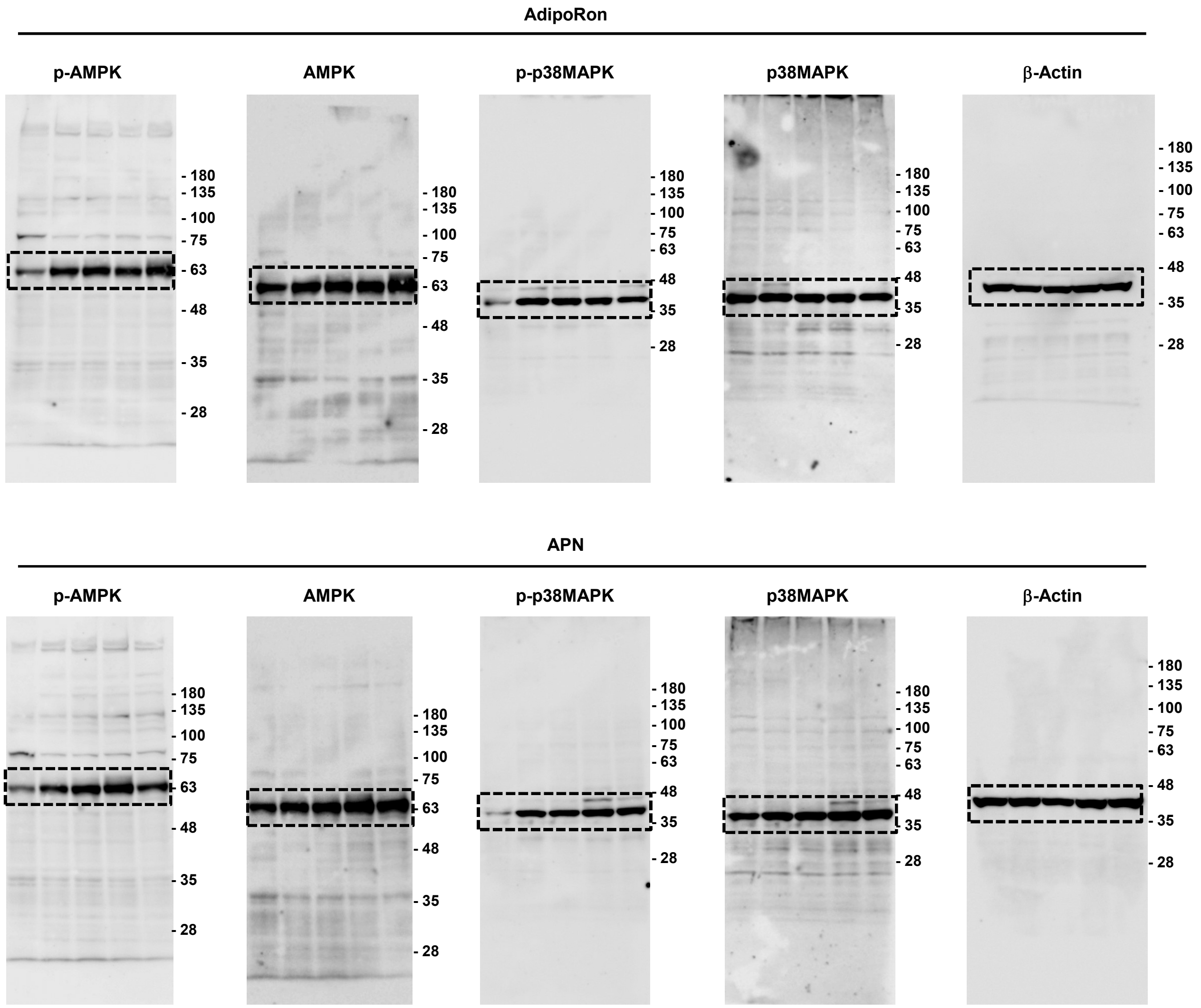
AdipoRon
p-AMPK
AMPK
p-p38MAPK
p38MAPK
β-Actin
APN
p-AMPK
AMPK
p-p38MAPK
p38MAPK
β-Actin

Fig. 6G

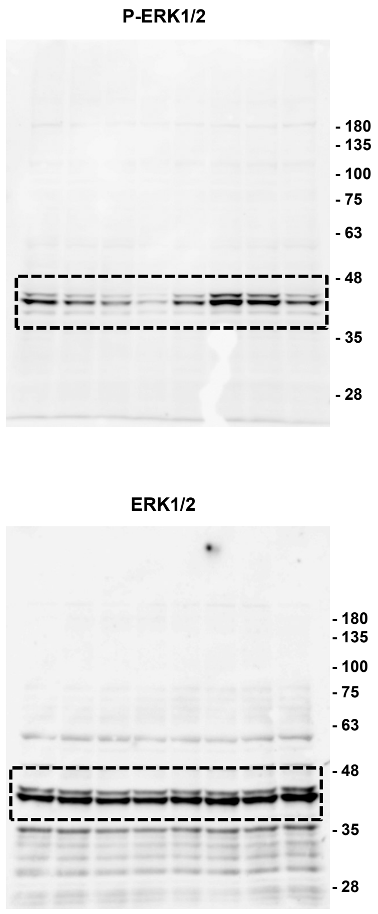
P-ERK1/2
ERK1/2

Fig. S2C

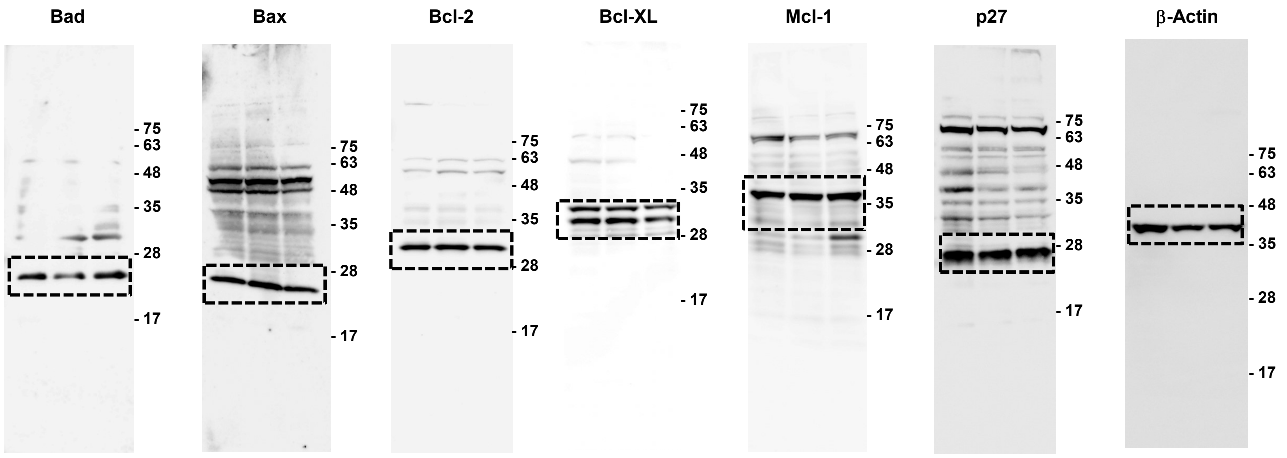

Fig. S8A

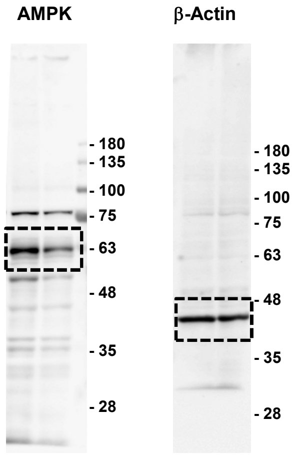

Fig. S10

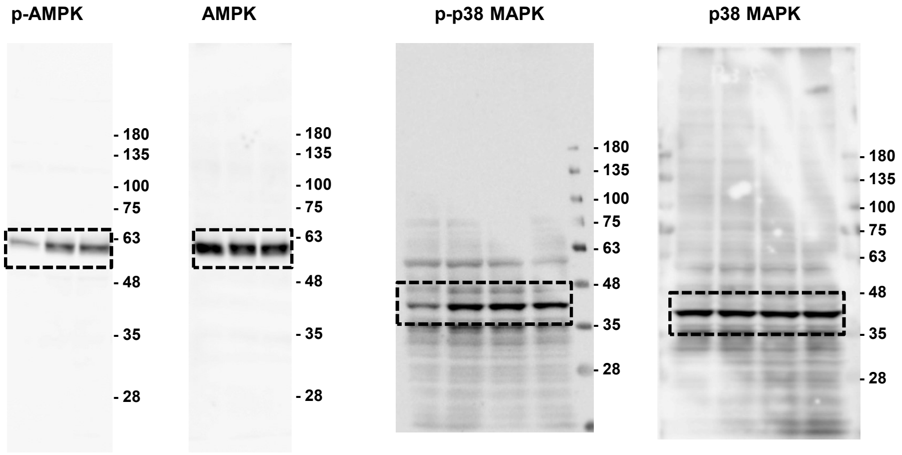

Fig. S13. Full size images of the Western blots presented in Figures.
