# Supplementary material for: Antidiabetic adiponectin receptor agonist AdipoRon suppresses tumour growth of pancreatic cancer by inducing RIPK1/ERK-dependent necroptosis
Source: Cell Death Dis. 2018 Jul 23;9(8):804. doi: 10.1038/s41419-018-0851-z (PMC6056513; doi:10.1038/s41419-018-0851-z)
Supplement: Supplementary file 2 — Table S1 [file 41419_2018_851_MOESM2_ESM.docx]

**Table S1. Primers used for real-time PCR.**

| **Primer name** | **Sequences (5' to 3')** | **Accession No.** | **Product**  **size (bp)** |
| --- | --- | --- | --- |
| GAPDH-F | CGCTCTCTGCTCCTCCTGTT | NM_002046.6 | 81 |
| GAPDH-R | CCATGGTGTCTGAGCGATGT |  |  |
| ADIPOR1-F | AATTCCTGAGCGCTTCTTTCCT | NM_015999.5 | 101 |
| ADIPOR1-R | CATAGAAGTGGACAAAGGCTGC |  |  |
| ADIPOR2-F | TGCAGCCATTATAGTCTCCCAG | NM_024551.2 | 101 |
| ADIPOR2-R | GAATGATTCCACTCAGGCCTAG |  |  |
| MCU-F | TACCACGTACGACCACCAAA | NM_138357.2 | 120 |
| MCU-R | TAACTGGTGCTGCTCAATGC |  |  |
| CAT-F | GCGGAGATTCAACACTGCCAATG | NM_001752.3 | 79 |
| CAT-R | CTGTTCCTCATTCAGCACGTTCAC |  |  |
| GPX3-F | GCCGGGGACAAGAGAAGT | NM_002084.4 | 131 |
| GPX3-R | GAGGACGTATTTGCCAGCAT |  |  |
| SOD1-F | ATGACTTGGGCAAAGGTGGAAATG | NM_000454.4 | 126 |
| SOD1-R | GTTAAGGGGCCTCAGACTACATCC |  |  |
| SOD2-F | CACATCAACGCGCAGATCAT | NM_000636.3 | 144 |
| SOD2-R | CAGTGCAGGCTGAAGAGCTATCT |  |  |
| UCP2-F | AGGGGCCCCGAGCCTTCTAC | NM_003355.2 | 113 |
| UCP2-R | GGCAGCCATGAGGGCTCGTT |  |  |
